# H19-Dependent Transcriptional Regulation of β3 and β4 Integrins Upon Estrogen and Hypoxia Favors Metastatic Potential in Prostate Cancer

**DOI:** 10.3390/ijms20164012

**Published:** 2019-08-17

**Authors:** Lorenza Bacci, Aurora Aiello, Cristian Ripoli, Rossella Loria, Dario Pugliese, Francesco Pierconti, Dante Rotili, Lidia Strigari, Francesco Pinto, Pier Francesco Bassi, Antonello Mai, Claudio Grassi, Alfredo Pontecorvi, Rita Falcioni, Antonella Farsetti, Simona Nanni

**Affiliations:** 1 Istituto di Patologia Medica, Università Cattolica del Sacro Cuore, 00168 Roma, Italy; 2 Institute of Cell Biology and Neurobiology, National Research Council, 00168 Rome, Italy; 3 Istituto di Fisiologia Umana, Università Cattolica del Sacro Cuore, 00168 Roma, Italy; 4 Fondazione Policlinico Universitario A. Gemelli IRCCS, 00168 Roma, Italy; 5 Department of Research Advanced Diagnostic and Technological Innovation, Regina Elena National Cancer Institute, IRCCS, 00144 Rome, Italy; 6 Dipartimento di Urologia, Università Cattolica del Sacro Cuore, 00168 Roma, Italy; 7 Dipartimento di Anatomia, Università Cattolica del Sacro Cuore, 00168 Roma, Italy; 8 Dipartimento di Chimica e Tecnologie del Farmaco, Sapienza Università di Roma, 00185 Roma, Italy; 9 Department of Medical Physics, S.Orsola Malpighi University Hospital, 40138 Bologna, Italy

**Keywords:** lncRNA, estrogen, hypoxia, prostate cancer, tumor metastasis, H19, epigenetic modulators, biomolecular analysis, targeted therapy

## Abstract

Estrogen and hypoxia promote an aggressive phenotype in prostate cancer (PCa), driving transcription of progression-associated genes. Here, we molecularly dissect the contribution of long non-coding RNA H19 to PCa metastatic potential under combined stimuli, a topic largely uncovered. The effects of estrogen and hypoxia on H19 and cell adhesion molecules’ expression were investigated in PCa cells and PCa-derived organotypic slice cultures (OSCs) by qPCR and Western blot. The molecular mechanism was addressed by chromatin immunoprecipitations, overexpression, and silencing assays. PCa cells’ metastatic potential was analyzed by in vitro cell-cell adhesion, motility test, and trans-well invasion assay. We found that combined treatment caused a significant H19 down-regulation as compared with hypoxia. In turn, H19 acts as a transcriptional repressor of cell adhesion molecules, as revealed by up-regulation of both β3 and β4 integrins and E-cadherin upon H19 silencing or combined treatment. Importantly, H19 down-regulation and β integrins induction were also observed in treated OSCs. Combined treatment increased both cell motility and invasion of PCa cells. Lastly, reduction of β integrins and invasion was achieved through epigenetic modulation of H19-dependent transcription. Our study revealed that estrogen and hypoxia transcriptionally regulate, via H19, cell adhesion molecules redirecting metastatic dissemination from EMT to a β integrin-mediated invasion.

## 1. Introduction

Prostate cancer (PCa) is one of the most common cancers in developed countries [1]. Widespread prostate-specific antigen (PSA) screening has revealed that most patients present localized disease [2], for which radical prostatectomy (RP) and radiation therapy (RT) are standard treatments. However, within 10 years, 20%–40% of post-RP [3] and 30%–50% of post-RT [4] patients will experience biochemical recurrence. On average, this precedes the appearance by eight years after RP of clinical metastases [5,6] that are the major cause of complications and death [7]. For these patients, a successful therapy is still unavailable, creating the need for additional prognostic markers and for molecular targeted therapeutical strategies.

The tumor microenvironment plays a crucial role in tumor initiation and progression. PCa is a hormone-dependent neoplasia in which ageing is a recognized risk factor. The decline in the androgen to estrogen ratio occurring with ageing is of key relevance for PCa development [8]. Estrogen/estrogen receptors signaling indeed plays a fundamental role in PCa carcinogenesis and progression [9,10]. Several evidences substantiated the contribution of both estrogen receptors (ERα or ERβ) and of non-genomic estrogen pathways in PCa, although there are conflicting results about the role of ERβ, commonly considered to play an anti-oncogenic role. In contrast to that, hormone naïve prostate cancer, unlike high grade prostate in situ neoplasia, generally retains high levels of ERβ expression, even in lymph node and bone metastasis [10], in favor of a pro-tumoral role, depending on cancer stage [11,12]. Of note, ERβ is extensively expressed in the secretory luminal cell types, in both benign and neoplastic lesions, as well as in prostate cancer stem cells.

A common feature of most solid tumors is a low oxygen tension, hypoxia, which induces proliferation, survival, apoptosis, metabolism, migration, and inflammation [13]. In particular, it is well established that both acute and chronic hypoxia co-exists in PCa providing a link between the hypoxic microenvironment and resistance to current therapy and poor outcome [14,15]. The critical molecular mediators of hypoxia, the hypoxia-inducible factors HIF-1α and HIF-2α, are stabilized upon low oxygen tension and transcriptionally regulate multiple steps of tumorigenesis [16]. Despite sharing similar structures and upregulation in many cancers, HIF-1α and HIF-2α do not appear to be fully redundant in function, raising the possibility that they may have non-equivalent effects in the neoplastic pathogenesis [17,18].

PCa progression and metastatization include alterations in epithelial–stroma interactions, induction of cell migration, and modulation of both extracellular matrix (ECM) and cell adhesion molecules [19,20]. Among the numerous cell surface and ECM molecules potentially involved, both integrins and cadherins are critical contributors to prostate cancer and represent potential therapeutic targets [21,22,23]. Integrins are transmembrane proteins consisting of heterodimeric α and β subunits that play an important role by bridging cells to ECM and by transmitting external signals to the cell nucleus, with a crucial role in tumor promoting exerted by laminin-binding integrins α3β1 and α6β4 [24], as well as by αvβ3 for bone metastasis [25]. Cadherins are cell surface proteins involved in homophilic cell–cell interactions influencing biological responses in complex ways [26]. The calcium-dependent E-cadherin (CDH1) is known to suppress tumorigenicity, and several mechanisms to impair CDH1 function were reported in cancer including epithelial to mesenchymal transition (EMT) [27]. Overall, dysregulation of integrins and cadherins triggers several signaling pathways determining cancer progression.

Numerous studies have shown the importance of long non-coding RNAs (lncRNAs) as modulators of key cellular physiological processes, such as scaffold for epigenetic modifying complex or as molecular sponge for miRNA [28], as well as in several pathologies such as cancer, including prostate cancer [29]. Emerging evidence indicates that the oncofetal H19 lncRNA plays a critical role during the complex process of tumorigenesis, starting from the early stages involving translational deregulation and genomic instability, through proliferation imbalance and stress management to metastasis [30]. The function of H19 is still controversial, exhibiting a promoting role in oncogenesis [31,32,33], but also acting as a tumor suppressor [34,35]. Thus, H19 may be capable of opposite functions largely dependent on cell type and microenvironment. Furthermore, H19 is one of the most sensitive lncRNAs to hypoxia [36] and estrogens [37]. Although evidence exists about the tumor suppressor role of H19 in metastatic PCa [29,38], the pathophysiological role of H19 in this cancer is not clearly elucidated. Zhu and colleagues have shown that the ectopic overexpression of H19 in metastatic prostate cell lines significantly increases the level of miR-675 and represses cell migration [38]. Nevertheless, little is known about a direct role of H19 itself in initiation and progression of PCa.

We previously demonstrated that signaling through ER, endothelial nitric oxide synthase (eNOS), and hypoxia inducible factors (HIFs), drives transcription of genes mediating acquisition of aggressive phenotype in PCa [39,40,41,42,43]. Among the non-coding RNAs modulated by estrogens in aggressive PCa [40,41], H19 genomic region was highly enriched in eNOS-peaks. Here, we focused our attention on the role of H19 in the molecular mechanisms by which estrogen and hypoxia signaling might favor the acquisition of an aggressive phenotype in prostate cancer. We showed that H19 is upregulated by estrogen or hypoxia, whereas it is reduced upon combined treatment. In turn, H19-dependent transcription of cells adhesion molecules and induction of E-cadherin and β3 and β4 integrins were observed upon combined treatment. Overall, these data reveal that combined stimuli switch tumor dissemination program from EMT to a β integrin-mediated invasion.

## 2. Results

### 2.1. Response of H19 Transcripts to Combined Estrogen and Hypoxia

Our previous data obtained by chromatin immunoprecipitation-sequencing (ChIP-Seq) showed that in response to estrogen, the transcriptional complex ER beta (ERβ) and eNOS is enriched on chromatin, as compared with the control, especially along non-coding regions [40,41]. Here, we focused on the H19 transcript, one of the lncRNAs most sensitive to estrogen [37] or hypoxia [36]. We found that H19 is significantly induced by hypoxia and by estrogens in hormone-driven tumor cells, such as breast and prostate cancer cells (Figure S1).

**Figure 1:**
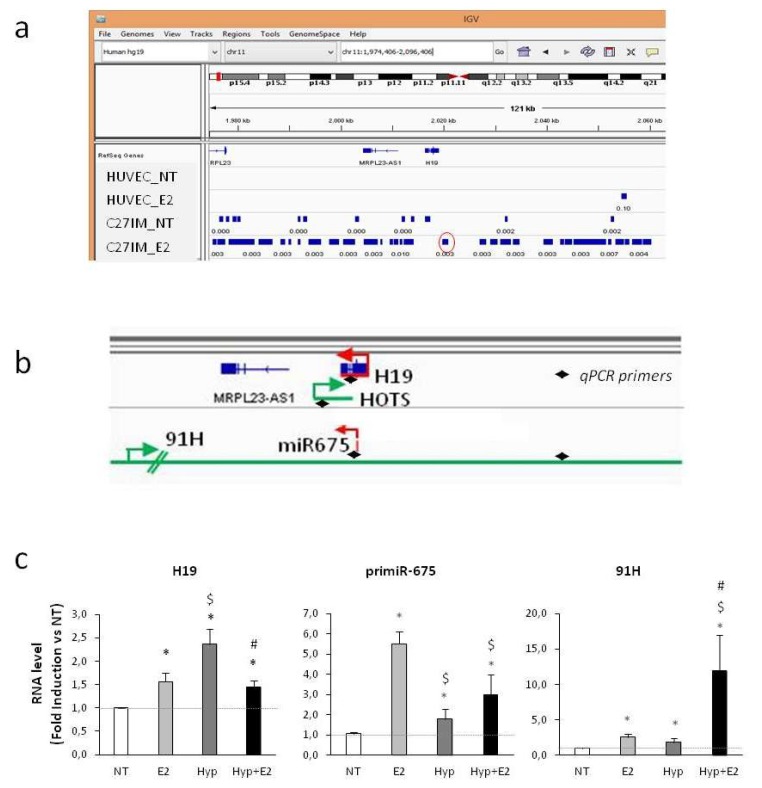
Response of H19 locus transcripts to estrogen and/or hypoxia in single or combined treatment. (**a**) Integrated Genome Viewer (IGV 2.1) screenshot showing H19 genomic region and endothelial nitric oxide synthase (eNOS) peaks (blue boxes) identified by chromatin immunoprecipitation (ChIP)-Seq in normal human primary umbilical vein endothelial cells (HUVECs) and prostate cancer C27IM cells, before and after 17 β-estradiol (10^−7^ M, E_2_). Red circle indicates the eNOS peak nearest to the H19 transcriptional start site. (**b**) H19 gene locus products, H19, miR-675, HOTS and 91H, and direction of transcription showed by green (sense) and red (antisense) arrows. Specific primers for qPCR are indicated with black arrowheads. (**c**) H19, primiR-675, and 91H RNA levels were assessed by quantitative RT-PCR in C27IM after 6 h treatment with estrogens (E_2_, 10^−7^ M) and 1% O_2_ hypoxia (Hyp) alone or in combination (18 h for primiR-675). Data, plotted as fold induction, represent mean ± SEM of three experiments. * *p* < 0.05 vs. NT; $ *p* < 0.05 vs. E_2_; # *p* < 0.05 vs. Hyp.

ChIP-Seq data (Figure 1a) showed that the H19 genomic region was highly enriched in eNOS-peaks, especially after 17β-estradiol (E2) treatment, and that these peaks were present in prostate epithelial luminal cells derived from aggressive primary cancer (C27IM, exclusively expressing beta isoform of the estrogen receptor [39,42,43]), but not in normal human primary umbilical vein endothelial cells (HUVECs). The H19 gene locus is a complex region; it can be transcribed in both sense and antisense directions (Figure 1b), producing from one strand H19 and the miR-675, embedded in H19’s first exon, and from the opposite strand, the transcript coding the HOTS protein and the antisense 91H. We focused on the response to estrogen and hypoxia of the non-coding transcripts, H19, miR-675, and 91H, in aggressive PCa cells. H19 was induced by hypoxia (1% O_2_, Hyp) or estrogen (10^−7^ M, 17β-estradiol, E_2_) at 6 h of treatment, a timing based on the previous time course for combined treatment [39]. Importantly, estrogen plus hypoxia (Hyp+E_2_) caused a significant downregulation of H19 expression as compared with hypoxia alone (Figure 1c). The primiR-675 was also downregulated, whereas the antisense transcript 91H was induced (Figure 1c).

To understand whether the H19 downregulation was specific for aggressive PCa, H19 expression was evaluated in normal cell lines (HUVEC), in cells derived from non-aggressive PCa (C38IM), and in metastatic PCa cell lines (PC3). As shown in Supplementary Figure S2, in HUVECs, the H19 level was not altered by estrogen or hypoxia, alone or in combination, while in C38IM, it was induced by hypoxia alone, but not altered by estrogen in combination. On the contrary, in the metastatic cell line PC3, a significant H19 downregulation was observed upon combined treatment as compared with hypoxia alone. These data suggest a specific downregulation of H19 expression upon combined treatment at least in aggressive prostate cancer cells (C27IM and PC3).

To corroborate these findings, we investigated the response of the H19 gene products to chemical hypoxia using cobalt chloride (100 µM, CoCl_2_). As shown in Supplementary Figure S3, H19 and primiR-675 were downregulated in C27IM under combined chemical hypoxia plus estrogen treatment, while the antisense transcript 91H was upregulated. Remarkably, this upregulation upon the double stimuli is in agreement with the oncogenic function of 91H reported in several tumors [44]. Furthermore, it is in agreement with the well-known regulation of classical hypoxia and estrogen target genes, such as the vascular endothelial growth factor receptor 2 (KDR, Figure S3d) and erythropoietin (EPO, Figure S3e), which exert a driving role in disease progression [39].

### 2.2. Transcriptional Regulation of H19 upon Combined Treatment

**Figure 2:**
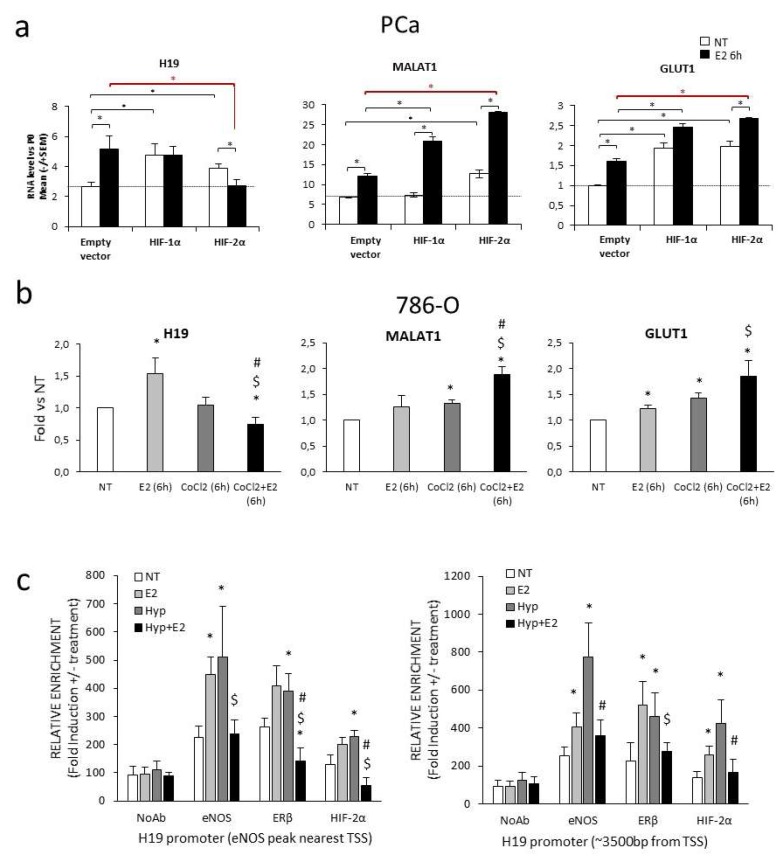
Transcriptional regulation of H19 upon estrogen, chemical hypoxia, or hypoxia in single or combined treatment. (**a**) C27IM cells were transfected for 72 h with hypoxia inducible factor (HIF)-1α or HIF-2α expression vectors. The empty vector Puc18 (empty vector) was used as control. H19, MALAT1, and GLUT-1 levels were quantified by qPCR in presence or absence of E_2_ (10^−7^ M; 6 h). Data represent mean ± SEM of three experiments. * *p* < 0.05. (**b**) H19, MALAT1, and GLUT1 levels were quantified by qPCR in human renal cancer cell line (786-O) after 6 h treatment with E_2_ (10^−7^ M) and CoCl_2_ (100 µM) alone or in combination. Data, plotted as fold induction, represent mean ± SEM of three experiments. * *p* < 0.05 vs. NT; $ *p* < 0.05 vs. E_2_; # *p* < 0.05 vs. CoCl_2_. (**c**) Recruitment on H19 promoter regions, at the eNOS-peak outlined with a red circle in Figure 1a (left) and about 3500 bp from the transcriptional start site (TSS) (right), of eNOS, ERβ, and HIF-2α by ChIPs after 2 h 15 min treatment with estrogen (E_2_, 10^−7^ M) and 1% O_2_ hypoxia (Hyp), alone or in combination, in prostate cells. No antibody (NoAb) served as the negative control. Values represent mean of three independent experiments. * *p* < 0.05 vs. NT; $ *p* < 0.05 vs. E_2_; # *p* < 0.05 vs. Hyp.

To understand the molecular mechanisms underlying the H19 downregulation upon combined stimuli, we investigated H19 transcription by parallel overexpression of HIF-1α or HIF-2α in the presence or absence of estrogen (E_2_) in PCa cells (Figure 2a, Figure S4). In the absence of overexpression (empty vector), E_2_ treatment significantly induced H19 expression (about 2-fold). Transfection of exogenous HIF-1α or HIF-2α (white bars in Figure 2a, left panel) resulted in increasing H19 basal expression, whereas estrogen treatment repressed the H19 level exclusively upon HIF-2α overexpression as compared with control (empty vector plus estrogen treatment, black bars in Figure 2a, left panel). Of note, levels of MALAT1, the well characterized lncRNA reported as a HIF-2α target [45], increased upon HIF-2α, but not HIF-1α overexpression (Figure 2a, middle panel). Meanwhile, in the presence of estrogen, it further increased, regardless of exogenous HIFs. Moreover, the hypoxia-target gene GLUT1 was induced, as expected, by both HIF-1α or HIF-2α overexpression and by estrogen (Figure 2a, right panel).

To confirm the selective involvement of HIF-2α, but not that of HIF-1α, in the H19 downregulation, we evaluated changes in the H19 response to combined treatment in the VHL-deficient renal carcinomas cells (786-O) that express endogenous HIF-2α, but not HIF-1α [46,47]. In this cellular context, H19 levels decreased upon the combined treatment as compared with hypoxia or estrogen alone, whereas MALAT1 and GLUT1 were induced under the same experimental condition (Figure 2b).

To corroborate the above findings, modulation of H19 in 786-O cells upon single or combined treatment was evaluated before and after HIF-2α silencing by small interfering RNA (Figure S5a). Notably, HIF-2α silencing was capable of rescuing H19 transcript upon combined treatment in the HIF-2α^+^ VHL-deficient renal carcinomas cellular context (Figure S5b). Similar results were obtained also in C27IM cells (Figure S5c,d). Of note, a strong H19 increase in basal condition was observed upon siHIF-2α in both cell lines, suggestive of a repressive role of HIF-2α in H19 regulation.

In addition, the H19 promoter was analyzed in C27IM by chromatin immunoprecipitation [42] (ChIP) under individual or combined stimulus (estrogen and hypoxia). We previously observed that several genes associated with progression were transcriptionally co-regulated by ERβ, HIFs, and eNOS under combined treatment [39]. Chromatins were immunoprecipitated by antibodies to ERβ, HIF-2α, or eNOS and DNA sequences proximal to or encompassing estrogen and hypoxia response elements within H19 regulatory sequences were amplified. We focused first on the eNOS peak identified by ChIP-Seq nearest to the transcriptional start site (TSS) and containing the previously reported hypoxia responsive elements (red circle in Figure 1a and the work of [31]). Induction of eNOS, ERβ, and HIF-2α recruitment was observed in response to estrogen or hypoxia. The combined treatment, as compared with the single stimulus, caused a significant reduction in eNOS, ERβ, and HIF-2α recruitment at the eNOS peak (Figure 2c, left). Similar results were obtained at the canonical estrogen responsive elements located about −3500 bp from TSS (Figure 2c, right). These data suggest that both ERβ or HIF-2α interact with chromatin at the estrogen and/or hypoxia responsive elements onto H19 promoter, whereas eNOS acts as a partner of the combinatorial complex (according to previous results [39,40,41,43,48]). Mechanistically, decreased recruitment of eNOS, ERβ, and HIF-2α and inhibition of H19 under the combined treatment may be linked to the repressive complexes like histone deacetylases HDACs [41]. In line with this, the addition of a specific inhibitor of HDAC1/2 (Mocetinostat) [49] determined an increase in the H19 level under combined treatment compared with the control (Figure S6).

### 2.3. Cell Adhesion and Invasion Modulation Upon Combined Treatment

**Figure 3:**
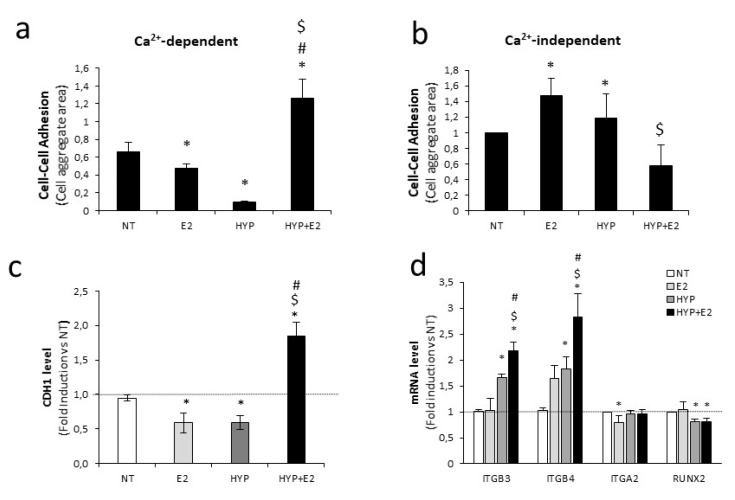

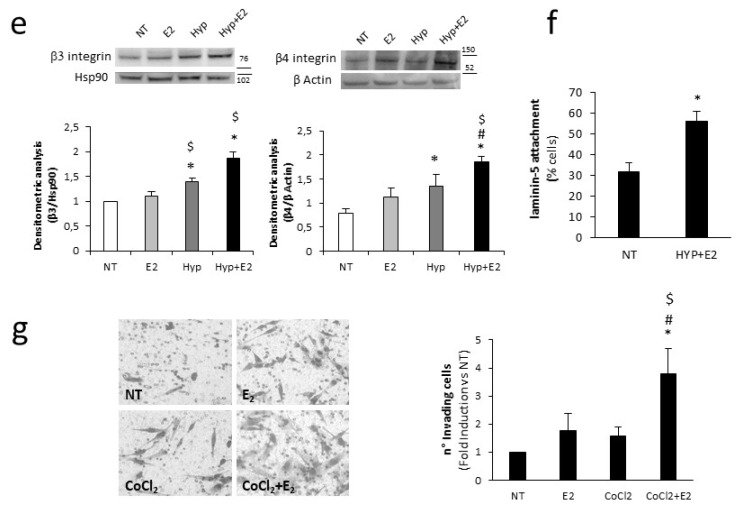
Cell adhesion and invasion modulation upon single or combined treatment. (a,b) Cell–cell adhesion assays were performed in the presence (Ca^2+^-dependent) or in absence (Ca^2+^-independent) of 1mM Ca^2+^ using cells cultured for 18 h with E_2_ (10^−7^ M) or 1% O_2_ hypoxia (HYP) alone or in combination (representative images in Figure S7a). Quantification of the in vitro cell–cell adhesion assay in the presence (a) or in absence (b) of 1mM Ca^2+^ is plotted as percentage of occupied area by cell aggregates. Data represent mean ± SEM of three independent experiments. (c) E-cadherin mRNA level was assessed by quantitative RT-PCR in C27IM after 18 h treatment with E_2_ (10^−7^ M), 1% O_2_ (HYP), alone or in combination. Data, plotted as fold induction, represent mean ± SEM of three experiments. (d) ITGB3, ITGB4, ITGA2, and RUNX2 mRNA level was assessed by quantitative RT-PCR in C27IM after 18 h treatment with E_2_ (10^−7^ M) or 1% O_2_ hypoxia (Hyp) alone or in combination (48 h for ITGB3). Data, plotted as fold induction, represent mean ± SEM of three experiments. (e) Protein level analysis of β3 and β4 integrins in C27IM after 18 h treatment with E_2_ (10^−7^ M) or 1% O_2_ hypoxia (HYP), alone or in combination (48 h for β3 integrin), performed by Western blot. Tubulin or β Actin served as control. Molecular weight marker is indicated. Upper panels: representative experiments. Lower panels: densitometric analysis, reported as fold induction vs. NT. (f) After a pre-treatment of 18 h under normoxia (NT) or estrogen (E_2_, 10^−7^ M) plus 1% O_2_ hypoxia (HYP+E2), cells were plated on laminin-enriched matrix for 20 min. The results are expressed as percentage of adherent cells on laminin-enriched matrix. Data represent mean ± SEM of three independent experiments. (g) Cell invasion was examined by Boyden chamber after a pre-treatment of 48 h under normoxia (NT) or estrogen (E_2_, 10^−7^ M) and hypoxia (100 µM CoCl_2_) alone or in combination. Left panel: representative phase contrast microscopic images under 20× magnification (bright field) of invading cells. Right panel: number of invading cells was presented, as fold induction vs. NT, as mean ± SEM of three independent experiments. * *p* < 0.05 vs. NT, $ *p* < 0.05 vs. E_2_, # *p* < 0.05 vs. HYP or CoCl_2_.

To investigate in vitro the metastatic potential of PCa cells, a cell–cell adhesion assay was first performed [50]. The combined treatment induced cells aggregation in the presence of calcium (Ca^2+^), compared with the control or single treatment (Figure 3a, Figure S7a), while reduced cell-adhesion in the absence of Ca^2+^ (Figure 3b, Figure S7a). According to published data [51,52], E-cadherin (CDH1) expression decreased upon estrogen or hypoxia, favoring EMT. In line with this, E-cadherin expression decreased, while other EMT-related genes, Snail, Vimentin, and N-cadherin, increased upon single stimuli (Figure 3c, Figure S7b,c). On the contrary, the combined treatment induced CDH1 expression at both the mRNA and protein levels, and reduced the EMT-related genes (Figure 3c, Figure S7b,c), as well as migration by scratch test (Figure S7d), thus suggesting that the EMT process is impaired in prostate cancer cells under estrogen plus hypoxia stress. Given that an interdependent network between cadherins and integrins has been proposed as crucial for the metastatic phenotype [53], we evaluated the expression of several integrin subunits in our experimental setting. Of note, combined treatment increased the β integrin subunits at the mRNA and protein levels, specifically β3 and β4 integrin (ITGB3 and ITGB4, Figure 3d,e), known to preferentially mediate the interaction with vitronectin and laminin 5 of the basal membrane, respectively. No modulation of the α2 integrin subunit gene (ITGA2) or in the unrelated gene Runt-related transcription factor 2 (RUNX2) was instead observed (Figure 3d). Moreover, the Laminin 5-enriched matrix cell-motility test revealed that combined treatment increased cell adhesion and motility as compared with the control (Figure 3f). Of note, the overall aggressiveness of PCa cells was highly induced by combined treatment, as revealed by an invasion test on Boyden chamber (Figure 3g).

### 2.4. H19 Mediates Transcriptional Repression of Cell Adhesion Molecules

**Figure 4:**
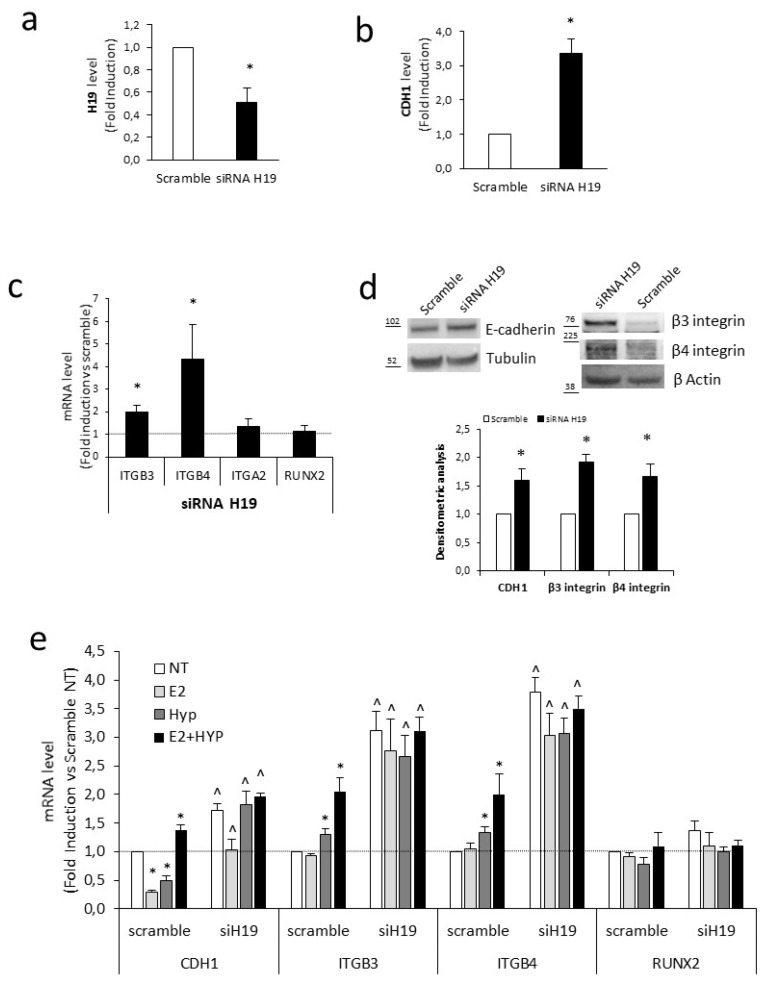
H19 mediates transcriptional repression of cell-adhesion molecules. (**a–c**) H19 (**a**); E-cadherin (CDH1) (**b**); ITGB3, ITGB4, ITGA2, and RUNX2 (**c**) mRNA levels quantified by qPCR in C27IM transfected with siRNA specific to H19 (siRNA H19) or scramble. Data, plotted as fold induction siH19 vs. scramble, represent mean ± SEM of three experiments. * *p* < 0.05 vs. scramble. (**d**) Protein analysis of E-cadherin, β3, and β4 integrin before and after siRNA H19 by Western blot. Tubulin or β Actin served as control. Molecular weight marker is indicated. Upper panels: representative experiments. Lower panel: densitometric analysis, reported as fold induction vs. scramble, represent mean ± SEM of three experiments. * *p* < 0.05 vs. scramble. (**e**) CDH1, ITGB3, ITGB4, and RUNX2 mRNA levels quantified by qPCR in C27IM transfected with siRNA specific to H19 (siH19) or scramble for 48 h and treated for additional 18 h with E_2_ (10^−7^ M), 1% O_2_ (HYP), alone or in combination. Data, plotted as fold induction vs. scramble NT, represent mean ± SEM of three experiments. * *p* < 0.05 vs. scramble NT, ^ *p* < 0.05 vs. scramble.

In bladder cancer cells, it has been reported that (i) the formation of a complex between H19 and the polycomb subunit EZH2 directly inhibits CDH1 expression by modulation of trimethylated lysine 27 of histone H3 (H3K27me3) level onto the CDH1 promoter [51], and (ii) a reduction of ITGB3 mRNA occurs upon H19 overexpression [54]. We asked whether H19 is involved in cell-adhesion molecules’ regulation in the PCa context. H19 silencing by small interfering RNA (Figure 4a) determined an increase of CDH1 as well as ITGB3 and ITGB4 mRNAs and protein level (Figure 4b–d). On the contrary, expression of ITGA2 and RUNX2 was unchanged (Figure 4c). Accordingly, H19 silencing increased the level of both ITGB3 and ITGB4 in the basal condition and further increased CDH1, ITGB3, and ITGB4 levels upon single or combined treatment, whereas RUNX2 expression was unaffected (Figure 4e). This suggests H19 acting as a transcriptional repressor of specific cell adhesion molecules.

**Figure 5:**
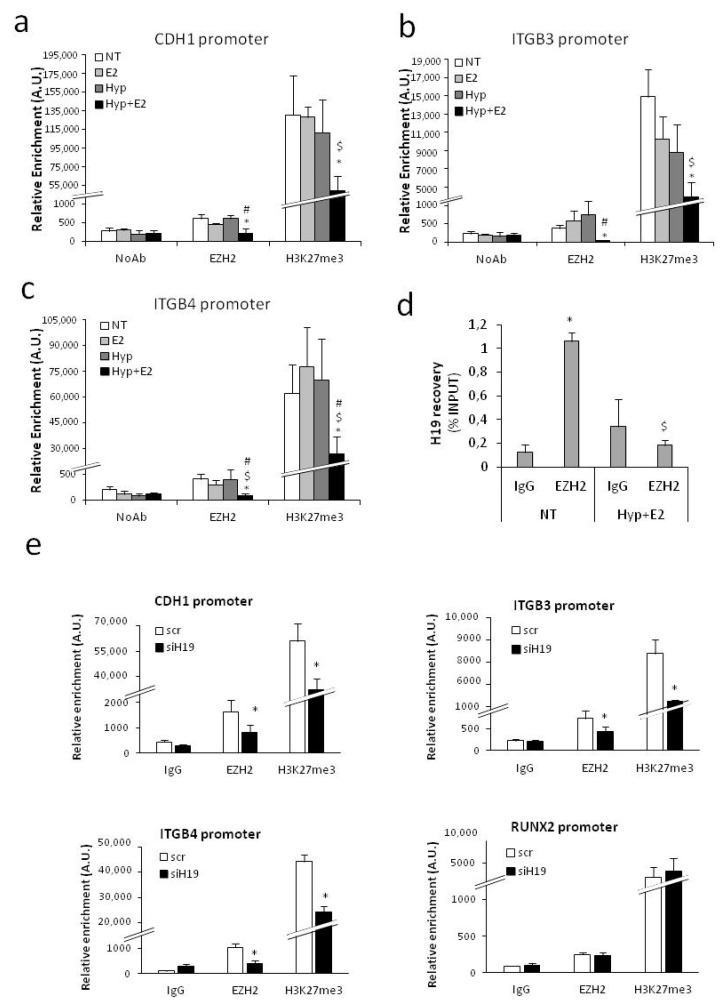
EZH2 recruitment and epigenetic modifications onto promoter of cell-adhesion molecules upon combined treatment or H19 silencing. (**a**–**c**) Recruitment of EZH2 and H3K27me3 level on the promoter region of CDH1 (**a**), ITGB3 (**b**), and ITGB4 (**c**) by ChIPs after 18 h treatment with estrogen (E_2_, 10^−7^ M), 1% O_2_ hypoxia, alone or in combination in PCa cells (C27IM). No antibody (NoAb) served as negative control. Values represent mean ± SEM of three independent experiments. * *p* < 0.05 vs. NT, $ *p* < 0.05 vs. E_2_, # *p* < 0.05 vs. Hyp. (**d**) In vivo H19 interaction with polycomb subunit EZH2 before and after Hyp+E2 (18 h) detected by RNA-ChIP assays. IgG was used as control. Immunoprecipitated RNA was recovered and analyzed by qRT-PCR. The results are expressed as mean ± SEM of three independent experiments. * *p* < 0.05 EZH2 vs. IgG, $ *p* < 0.05 Hyp+E2 vs. NT. (**e**) Recruitment of EZH2 and H3K27me3 level on the promoter region of CDH1, ITGB3, ITGB4, and RUNX2 by ChIPs upon H19 silencing (siH19) compared with control (scamble, scr) in PCa cells (C27IM). Non-specific immunoglobulin (IgG) served as negative control. Values represent mean ± SEM of three independent experiments. * *p* < 0.05 vs. scramble.

In line with this, the recruitment of EZH2 and level of H3K27me3 onto CDH1 as well as on ITGB3 and ITGB4 promoters decreased upon combined treatment (Figure 5a–c). Interaction on chromatin between H19 and EZH2 in PCa cells was indeed confirmed by RNA-ChIP in the basal condition, whereas the combined treatment specifically impaired this interaction (Figure 5d). Accordingly, the recruitment of EZH2 and level of H3K27me3 onto CDH1 as well as on ITGB3 and ITGB4 promoters decreased upon H19 silencing. No changes were observed in the control gene promoter RUNX2 (Figure 5e).

### 2.5. H19 and Integrin Modulation on Ex Vivo Organotypic Slice Cultures (OSCs) of Prostate Tumor upon Combined Treatment

**Figure 6:**
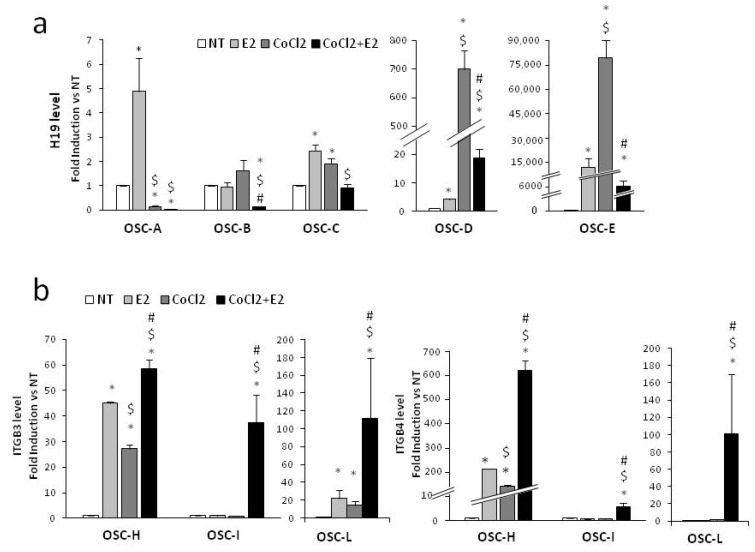
H19 and integrin modulation on ex vivo PCa-derived organotypic slice cultures (OSCs) upon single or combined treatment. (**a**) H19 levels were assessed by qPCR in OSCs from five different PCa patients (OSC A–E) after 6 h treatment with E_2_ (10^−7^ M) and CoCl_2_ (300 µM), alone or in combination. Data are plotted as fold induction vs. NT. (**b**) ITGB3 and ITGB4 levels were assessed by qPCR in OSCs from three different PCa patients after 48 h treatment with E_2_ (10^−7^ M) and CoCl_2_ (300 µM), alone or in combination. Data are plotted as fold induction vs. NT. * *p* < 0.05 vs. NT, $ *p* < 0.05 vs. E_2_, # *p* < 0.05 vs. CoCl_2_.

To evaluate whether the H19 downregulation observed in PCa cells was also detectable in tissue samples from patients with prostate cancer (*n* = 10), a molecular analysis was performed using organotypic slice cultures (OSCs) obtained from fresh surgical explants of organ-confined prostate tumors, as in the work of [40] (Figure S8 and Table 1 for histopathological characteristics of PCa patients). H19 expression was assessed in OSCs treated with 300 µM CoCl_2_. This concentration was adopted because it was capable of inducing the hypoxia responsive gene GLUT1 (Figure S8b), alone or in combination with estrogen. Seven OSCs were treated for 6 h with E_2_ and/or CoCl_2_ and H19 detected in five out of seven samples. Remarkably, a significant H19 downregulation was observed upon combined treatment, as compared with single stimulus in all samples (Figure 6a). In order to evaluate integrins expression under combined treatment, a subset of OSCs (*n* = 3) was treated with estrogen and hypoxia for a longer time (48 h). As shown in Figure 6b, a significant increase of ITGB3 mRNA was detected in all samples upon individual and combined treatment, with the exception of OSC-I being virtually responsive to the combined treatment alone. ITGB4 mRNA was responsive to single treatment only in OSC-H, whereas combined estrogen and CoCl_2_ was effective in all three OSCs. Note that the increase of β4 integrin was also observed at the protein level in OSC-L (Figure S8c). These results, although very limited because of the small number of samples, show, at least as proof of principle, that estrogen counteracts hypoxia in terms of H19 modulation also in the ex vivo PCa samples, and are in agreement with previous data obtained in PCa cell lines.

### 2.6. Effects of Epigenetic Drugs on H19/Integrin Pathway upon Combined Treatment

**Figure 7:**
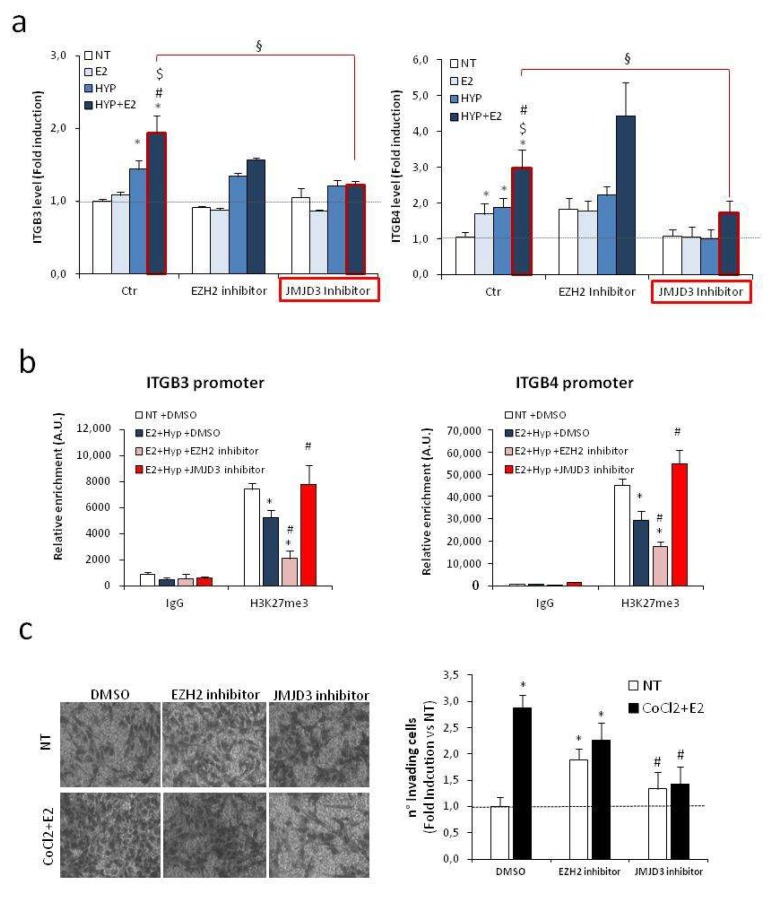
Effects of epigenetic drugs on H19/integrin pathway. (**a**) ITGB3 (left) and ITGB4 (right) mRNA levels were assessed by qPCR in C27IM after 18 h treatment with estrogen (E_2_; 10^−7^ M), 1% O_2_ hypoxia (HYP), alone or in combination, in the presence or absence of inhibitor specific to EZH2 (GSK-126, 1 µM) or JMJD3 (GSK-J4, 1 µM) added 30 min before E_2_ and/or HYP. Data, plotted as fold induction vs. Ctr/NT, represent mean ± SEM of three experiments. *p < 0.05 vs. NT, $ *p* < 0.05 vs. E_2_, # *p* < 0.05 vs. HYP, § *p* < 0.05 vs. HYP+ E_2_. (**b**) H3K27me3 level on the promoter region of ITGB3 and ITGB4 by ChIPs in C27IM cells treated as in panel a. Data represent mean ± SEM of three experiments. IgG served as negative control. * *p* < 0.05 vs. NT, # *p* < 0.05 vs. Hyp. (**c**) Cell invasion was examined by Boyden chamber after a pre-treatment of 48 h under normoxia (NT) or combination of estrogen (E_2_, 10^−7^ M) and hypoxia (100 µM CoCl_2_), in the presence or absence of EZH2 or JMJD3 inhibitor added as in (**a**). Vehicle (DMSO) was used as control. Left panel: representative phase contrast microscopic images under 20× magnification (bright field) of invading cells. Right panel: number of invading cells, presented as fold induction vs. NT DMSO, was mean ± SEM of three independent experiments. * *p* < 0.05 vs. NT DMSO, # *p* < 0.05 vs. CoCl_2_ + E_2_ DMSO.

We hypothesized that specific epigenetic drugs capable of affecting the H19/EZH2/H3K27me3 circuitry might contribute to normalizing the level of β integrin subunits under combined stimuli. To this end, PCa cells were treated with estrogen plus hypoxia in the presence or absence of the EZH2 or H3K27 demethylase JMJD3 specific inhibitors GSK-126 [55] and GSK-J4 [56], respectively. As control, the level of the known EZH2-target gene, CDH1 and cyclin D1 [57], was increased and decreased, respectively, with GSK-126 treatment (Figure S9). Treatment with GSK-J4, but not with GSK-126, specifically normalizes the levels of both ITGB3 and ITGB4 (Figure 7a). Of note, an analysis of H3K27me3 level on β3 and β4 integrins revealed that reduction observed upon combined stimuli compared with control was further decreased in the presence of EZH2 inhibitor GSK-126. Conversely, treatment with JMJD3 inhibitor GSK-J4 under estrogen plus hypoxia restored the basal level of H3K27me3 on the promoters analyzed (Figure 7b). Notably, treatment with GSK-J4, but not GSK-126, specifically inhibited PCa cells’ invasion by Boyden assays (Figure 7c).

These data suggest that blocking H19 action on integrins transcription may impair metastatic potential upon combined treatment, providing evidence for a potential novel targeted therapy in prostate cancer.

## 3. Discussion

Deciphering the mechanisms contributing to PCa metastatic progression is essential for discovering therapeutic strategies. The main finding of our study resides on the downregulation of H19 under combined estrogen and hypoxia stimuli, leading to induction of CDH1 and β3 and β4 integrin subunits, a mechanism that profoundly changes and affects PCa metastatic potential.

Our previous studies in prostate cancer revealed that estrogen—and hypoxia—signaling act synergistically to promote a specific gene transcription program towards the acquisition of an aggressive phenotype [39]. Here, in agreement with the literature, we showed that H19 was induced by hypoxia and by estrogen as single stimulus [36,37]. On the contrary, the combined treatment, hypoxia plus estrogen, impaired H19 expression (Figure 1, Figures S2 and S3). These data were unpredictable because H19, as with other cancer-associated lncRNAs, was expected to respond in a synergistic manner upon the pro-tumor stimuli, hypoxia and estrogen, in line with previous results on classical estrogen receptor and HIFs target genes, for example, KDR and EPO (Figure S3 and the work of [39]).

Mechanistically, H19 down-modulation upon combined stimuli appears to involve HIF-2α, but not HIF-1α. HIF-2α silencing, in fact, was capable of rescuing H19 transcription in that experimental condition (Figure S5). Moreover, recruitment of eNOS, ERβ, and HIF-2α was impaired upon combined treatment along H19 regulatory regions, specifically at two relevant genomic loci (Figure 2c). The relevance of a specific HIF-2α contribution is in line with previous results regarding the decrease of survival in PCa patients with a higher level of nuclear eNOS and HIF-2α [39]. Here, we hypothesize that one specific action of HIF-2α, at least in aggressive PCa, might be a direct inhibition of H19 transcription. Regarding HIF-1α, our data suggest that H19 downregulation upon combined treatment does not require HIF-1α function, as found in PCa and 786-O cells (Figure 2). These results are in line with the previous observation about HIFα isoforms’ specific transcriptional selectivity [17,58]. Molecularly, reduction of eNOS, ERβ, and HIF-2α recruitment and inhibition of H19 under combined treatment may be the result of post-translation/conformation changes of proteins altering protein–protein interaction with the transcriptional complexes. In this regard, based on our previous results on transcriptional regulation of miR-34a in aggressive PCa [41], we found that inhibition mediated by estrogens requires HDACs recruitment on target promoter. We also demonstrated the involvement of KMD1A and KMD4 demethylases in estrogen-mediated activation of hormone-target genes [40]. Moreover, emerging evidence has indicated a small number of histone methyltransferases (HMTs) and histone demethylases (HDMs) as regulators of ER signaling [59]. Here, we found that at least HDAC1/2 may affect the level of H19 under combined treatment (Figure S6), advocating the contribution of specific repressive complexes in H19 transcription regulation.

Some studies provided evidence suggesting that estrogen and hypoxic conditions *per se* can trigger an EMT program, potentiating the invasive capacity of a variety of cancers [60,61], and that estrogen-mediated signaling can either negatively or positively affect the hypoxia pathway, depending on cell type [59,62]. Our finding about E-cadherin downregulation and upregulation of EMT-related genes under hypoxia and estrogen alone is in agreement with induction of EMT in these experimental conditions, whereas upon combined stimuli, we observed a specific upregulation of E-cadherin, suggesting an EMT impairment (Figure 3c, Figure S7). Interestingly, E-cadherin increase was paralleled by upregulation of the β3 and β4 integrin subunits upon combined treatment (Figure 3d,e, Figure S7). The involvement of integrins in cell migration and spreading is well established [20], suggesting that upon combined stimuli, changes in the cell-adhesion molecules’ pattern may promote PCa aggressiveness via a mechanism residing in beta integrin-mediated motility, rather than EMT. This conclusion is supported by our results on laminin 5-motility, invasion assays, and scratch tests (Figure 3, Figure S7). Our data are also in line with previous findings demonstrating a role for integrin β3 in PCa bone metastatization [25] and an overexpression of β4 integrin in advanced human prostate cancer [63], and its association with aggressive behaviour and poor prognosis in several cancer types [64].

The co-expression of CDH1 and β integrins under combined stimuli is, moreover, in agreement with the recently described cohesive metastatic phenotype in human prostate cancer [65]. Evidence on PCa tissues revealed that aggressive PCa proceeds through a collective migration, a cluster of invasive cells, rather than a single cell migration. Importantly, the cluster cells require both laminin-binding integrins expression to ECM remodelling during migration, and cadherin expression to cell–cell cohesion supporting collective migration (hybrid epithelial/mesenchymal phenotype). Our data in PCa cells showed that combined estrogen and hypoxia treatment favours acquisition of both epithelial cell–cell adhesion and mesenchymal motility traits. Here, we showed that change in cell-adhesion molecule pattern is mediated by H19 function. Although previous data reported that H19 overexpression reduced ITGB3, ITGB5, and ITGA5 in bladder cancer cells [54] and increased ITGB1 and ITGA1 in pancreatic cancer cells [66], the molecular mechanism by which H19 regulates integrin was not elucidated. Here, we provided evidence in favor of H19 acting as a repressor of transcription of several cell-adhesion molecules, like CDH1, ITGB3, and ITGB4. Mechanistically, we demonstrated that H19, by forming a complex with the EZH2, modulates H3K27me3 level and ultimately the rate of transcription (Figure 5). In line with this, H19 silencing induced CDH1, ITGB3, and ITGB4 in the basal condition, as well as upon single or combined treatment (Figure 4). At the same time, it reduced EZH2 recruitment and H3K27me3 level on target promoters (Figure 5). Moreover, although several reports exist about estrogen or hypoxia as individual stimuli capable to induce beta integrin subunits [66,67,68,69,70], this is the first time, to the best of our knowledge, that an increase in beta integrin subunits is reported upon combined estrogen and hypoxia.

The results on PCa cells are also supported by data obtained in the ex vivo OSC model (Figure 6, Figure S8), showing that, also in PCa fresh tissue explants derived from patients, the combined treatment causes a specific H19 downregulation paralleled by β integrin subunits induction, a phenotype that could be used as biomarker to define a specific subset of PCa patients. On the basis of these preliminary data, we hypothesize that lower levels of basal H19 may be associated with the presence of both hypoxic area and estrogens stimuli in prostatic tumors that, in turn, mediate an H19-dependent beta integrin subunit induction. Further experiments will be required to investigate better this aspect.

**Figure 8:**
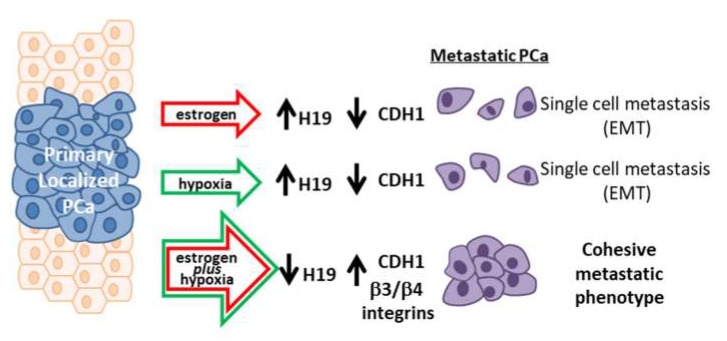
Cartoon of H19-dependent metastasis dissemination under combined treatment. Cartoon illustrating different mechanisms of metastasis dissemination, in which H19 is involved in aggressive prostate cancer under single or combined estrogen plus hypoxia stimuli.

Regarding the specific function of H19 in prostate cancer, an inhibitory role in cell migration for the H19 internal microRNA, miR-675, has been identified in metastatic prostate cancer cell line by Zhu and colleagues [38]. This activity as tumor suppressor is apparently in contrast with the upregulation of H19 observed under estrogen or hypoxia alone (Figure 1, Figures S2 and S3). At the same time, it appears to be in line with our results in terms of H19 downmodulation observed under combined treatment and with the known key role played by hypoxia and estrogen in tumor aggressiveness. Our data suggest that H19 may exert a different function in prostate cancer depending on tumor properties and tumor microenvironment. Under hypoxia or estrogen alone, H19 level increases and, through reduction of CDH1, induces EMT, exhibiting oncogenic properties. On the contrary, under combined stimuli, H19 level is transcriptionally reduced, and CDH1 and β integrin expression released, switching tumor dissemination toward an alternative mechanism, the cohesive phenotype, suggesting a tumor suppressor role for H19 in this condition (Figure 8).

The correlation between H19 and integrins and their involvement in the acquisition of aggressive phenotype potentially identify the H19/integrin pathway as novel target for designing molecular therapy. In this regard, a specific effect of the JMJD3 inhibitor GSK-J4 in preventing ITGB3 and ITGB4 upregulation upon combined treatment was observed in PCa cells (Figure 7a). Notably, specific JMJD3 inhibitor was able to significantly reduce invasion upon combined stimuli (Figure 7c). Future experiments in pre-clinical models are necessary to evaluate the efficacy of JMJD3 inhibitors as anti-tumor agents.

## 4. Materials and Methods

### 4.1. Reagents and Antibodies

17β-Estradiol (E_2_), cobalt chloride (CoCl_2_) (Sigma Aldrich, St. Louis, MO, USA)). Mocetinostat, GSK-126, and GSK-J4 inhibitors were as in the literature [49,55,56]. Antibody to β4 integrin (450-11A) was as in the work of [71]. Antibodies: E-cadherin (GeneTex, Irvine, CA, USA; GTX100443), ERβ (L-20, Santa Cruz Biotechnology, Dallas, TX, USA; SC-6822, CWK-F12, DSHB), eNOS (BD Biosciences, Franklin Lakes, NJ, USA; BD610297), EZH2 (D2C9, Cell Signaling, Danvers, MA, USA; #5246s), H3K27me3 (Active Motif, Carlsbad, CA, USA; #39156 and #39155), HIF-2α (Novus Biologicals, Centennial, CO, USA; NB100-132), HIF-1α (Novus Biological NB100-105), N-cadherin (Thermo-Fisher, Waltham, MA, USA; #PA5-19486), integrin β3 (Genetex, Irvine, CA, USA; GTX111672), Snail (Genetex GTX125918), Vimentin (Bethyl, Montgomery, TX, USA; A301-620A-T), Laminin beta 1 (Abcam, Cambridge, UK; ab108536), Fibrillarin (Thermo Fisher MA3-16771), HSP90 (StressMarq, Victoria, BC, Canada; SPC-104), IgG (Normal Rabbit, Millipore, Burlington, MA, USA #12-370, Normal Rabbit, Bethyl #S120-101), Tubulin (DM1A, Abcam #ab7291), β Actin (Abcam).

### 4.2. Cells and Treatments

Prostate epithelial luminal cancer (PCa) cell lines (C27IM and C38IM), PCa-metastasis-derived PC3, HUVEC, MCF7, and MDA-MB-361 were cultured as in the work of [40]. Renal carcinoma cells 786-O (VHL/HIF-1α deficient) were grown as in the work of [39]. The rat bladder epithelial cell line 804G was grown as in the work of [71].

At least 72 h before experimental use, cells were switched to medium with hormone-deprived serum and treated with 17β-estradiol (E_2_, 10^−7^ M; Sigma) for the times indicated in figure legends. Mocetinostat2HBr (1 µM), EZH2 (GSK-126, 1µM) and JMJD3 (GSK-J4, 1 µM) inhibitors were added to cell cultures 30 min before estrogen and/or hypoxia treatments. Hypoxia condition was obtained by the chemical agent cobalt chloride (CoCl_2_, 100 µM) or by 1% O_2_, as in the work of [39].

### 4.3. RNA Extraction and Real Time PCR

RNA extraction, cDNA preparation, and real time PCR analysis were performed as in the work of [40] on QuantStudio 5 Real-Time PCR System (Applied Biosystems, Foster City, CA, USA) using SYBR Green quantification. The relative amount of each gene was measured as fold change using the 2^−ΔΔ*C*t^ method (β-Actin or P0 served as endogenous control). Expression of β-Actin, GLUT1, and primiR-675 were evaluated by TaqMan method (Applied Biosystems). Primers for MALAT1 was as in the work of [40]. Primers sequences were as follows: 91H 5′-GGCCCTGAATCAAACATCATG-3′ and 5′-TCACGCAGCGTTGCTCTCT-3′, CDH1 5′-CTGGGACTCCACCTACAGAAAGTT-3′ and 5′-CCAGAAACGGAGGCCTGAT-3′, EPO 5′-AGCCCAGAAGGAAGCCATCT-3′ and 5′-GAAAGTGTCAGTGATTGTTC-3′, H19 5′-TCAAGCCTGGGCCTTTGAAT-3′ and 5′-GGCTGATGAGGTCTGGTTCC-3′, ITGA2 and 5′-CAGCAATGTGGGAATCAGTATTACA-3′ and 5′-GGAAGGGCAGGGCTGAGT-3′, ITGB3 5′-GAAAACCCCTGCTATGATATGAAGAC-3′ and 5′-GTTAGCGTCAGCACGTGTTTG-3′, ITGB4 5′-TGAAGAGCTGCACGGAGTGT-3′ and 5′-GGTCCCTGAACATCTCGTCTGT-3′, KDR 5′-CCAGCAAAAGCAGGGAGTCT-3′ and 5′-TGGTAGCCGCTTGTCTGGTT-3′, P0 5′-TCGACAATGGCAGCATCTAC-3′ and 5′-ATCCGTCTCCACAGACAAGG-3′, RUNX2 5′-CCCGTGGCCTTCAAGGT-3′ and 5′-TGACAGTAACCACAGTCCCATCTG-3′, HIF1α 5′-GAACAAAACACACAGCGAAGCT-3′ and 5′-TGCAGTGCAATACCTTCCATGT-3′, HIF2α 5′- ATCAGCTTCCTGCGAACACA-3′ and 5′-CTTCGGCTTCGGACTCGTT-3′, CCND1 5′-CCGTCCATGCGGAAGATC-3′ and 5′-TGCAGGCGGCTCTTTTTC-3′.

### 4.4. Transient Transfection Assays

Expression vectors were as described in the work of [39]. Plasmids were transfected into PCa cell line using Lipofectamine RNAiMAX (Invitrogen, Carlsbad, CA, USA) according to the manufacturer’s instructions. Cells were grown in hormone-deprived serum for 72 h from transfection and treated with 17β-estradiol (E_2,_ 10^−7^ M; Sigma) for 6 h before harvesting for RNA extraction.

### 4.5. Chromatin Immunoprecipitation (ChIP)

ChIPs were performed as in the work of [40]. Briefly, DNA fragments were recovered and analyzed by qPCR using SYBR Master mix (Applied Biosystems, Foster City, CA, USA) with evaluation of dissociation curves on QuantStudio 5 Real-Time PCR System (Applied Biosystems). Standard curves were generated by serially diluting the input (5-log dilutions in triplicate). The specific sequences isolated by the immune-complexes were analyzed by qPCR in duplicate and the data obtained were normalized to the corresponding DNA input control. Data are represented as relative enrichment (fold induction over control ± treatment). Immunoprecipitations were performed using specific antibodies to eNOS (BD Biosciences, BD610297, Franklin Lakes, NJ, USA), ERβ (L-20, Santa Cruz Biotechnology #SC-6822, CWK-F12, DSHB, Dallas, TX, USA;), HIF-2α (Novus Biologicals NB100-132, Centennial, CO, USA), EZH2 (D2C9, Cell Signaling, Danvers, MA, USA), and H3K27me3 (Active Motif #39156 and #39155, Carlsbad, CA, USA). No antibody or IgG (Bethyl. #P120-101, Montgomery, TX, USA) were used as negative control. Primers sequences were as follows: H19prom (eNOS peak) 5′-GCCATGGCCCTTGATAGCTA-3′ and reverse 5′-TCCATATAGTCTCGACGTCATCCA-3′, H19prom (-3500bp) 5′-CCTGTGTTCTGAGGTGATCATGA-3′ and 5′-GGCCTTGTGGGAAACGATT-3′; ITGB3prom forward 5′-CGAGGCTCTTCATGGACCTATC-3′ and reverse 5′-ACCTACCTGTTGAGGGCAAGAC-3′, ITGB4prom forward 5′-TGACCTGAACACCCGTGGTA-3′ and reverse 5′-GCACTCGATGCCTTGTTACAGT-3′, CDH1prom forward 5′-CCGTGCAGGTCCCATAACC-3′ and reverse 5′-CATAGACGCGGTGACCCTCTA-3′, RUNX2prom 5′- GCTCTGCTCTACAAATGCCTTAACCT-3′ and 5′-TCCGGCATCCAGAAGGATATAG-3′.

### 4.6. RNA Interference

Small interference to H19 and to HIF-2α was obtained with TriFECTa Kit DsiRNA Duplex (Integrated DNA Technologies) transfected using Lipofectamine RNAiMAX (Invitrogen) according to the manufacturer’s instructions and as in the work of [72]. Scrambled universal negative control RNA duplex (NC1) was used. RNA was extracted after 48 h and chromatin was crosslinked after 18 h from transfection.

### 4.7. RNA-Chromatin Immunoprecipitation (RNA-ChIP)

RNA-ChIPs were performed as in the work of [40] using specific antibodies to EZH2 (D2C9, Cell Signaling #5246s) and IgG (Normal Rabbit, Millipore #12-370). The specific sequences isolated by the immune-complexes were analyzed by real time PCR using the one-step method with SYBR Green and specific primers (RNA-to-Ct 1 step kit; Applied Biosystems) and primer PCR assay H19 (#12004175 Bio-Rad, Hercules, CA, USA).

### 4.8. Protein Extraction and Western Blot

Total protein extracts were obtained using RIPA buffer and nuclear/cytosolic extracts were prepared as in the work of [39]. Western blot assay was performed using 40 μg of total extract and proteins were resolved by SDS-PAGE using 7.5% or 4%−12% gradient Invitrogen Precast gel (NuPage and MES buffer). The following primary antibodies were used: E-cadherin (1:2000, GeneTex GTX100443), β4 integrin 450-11A (1:1000), HIF-2α (1:500, Novus Biologicals, Centennial, CO, USA; NB100-132), HIF-1α(1:500, Novus Biological NB100-105), N-cadherin (1:5000, Thermo-Fisher PA5-19486), integrin β3 (1:1000, Genetex GTX111672), Snail (1:1000, Genetex GTX125918), Vimentin (1:1000, Bethyl A301-620A-T), Fibrillarin (1:1000, Thermo Fisher MA3-16771), Laminin beta 1 (1:1000, Abcam ab108536), Tubulin (1:5000, DM1A Abcam #ab7291), β Actin (1.1000, Abcam), and HSP90 (1:1000, StressMarq SPC-104C). Specific protein signals were revealed with ECL Prime (Amersham, GE Healthcare, Boston, MA, USA) and detected by UVIDOC (Eppendorf S.r.l., Hamburg, Germany). The intensity of each band was evaluated using the NIH Image J 1.8 software (National Institutes of Health, Bethesda, MD, USA).

### 4.9. Cell–Cell Adhesion Assay

Cell–cell adhesion assay was performed as described in the work of [50] with minor modifications. Briefly, after 18 h of 17β-estradiol (E_2_) and/or 1% O_2_ hypoxia treatment, a single-cell suspension was prepared with 3 mmol/L EDTA in PBS^−/−^. Cells (6.6 × 10^5^) were suspended in 1.6 mL complete medium with 1 mmol/L CaCl_2_ (for Ca^2+^-dependent cell–cell adhesion) or 3 mmol/L EDTA (for Ca^2+^-independent cell–cell adhesion) and plated on 60 mm bacterial Petri dishes and incubated at 37 °C on a shaking platform for 60–90 min. Phase contrast images (bright field) were taken using a phase-contrast microscope (AXIO microscope with AxioCam Erc5s, Zeiss- objective 10×) and the cell aggregated area was measured with NIH Image J 1.8 software (National Institutes of Health, Bethesda, MD, USA).

### 4.10. Scratch Test

Scratch assay was performed as described previously in the work of [72]. Images were obtained using a phase-contrast microscope (AXIO microscope with Axio Cam Erc5s, Zeiss- objective 10×, Heidelberg, Germany) at time 0 and after 24 h, distance between edges was measured by the phase contrast microscopy and analyzed by ZEN imaging software (Zeiss, Heidelberg, Germany). Data were represented as percentage of the original gap.

### 4.11. Laminin 5 Cell Motility Test

A laminin 5-enriched matrix was prepared from 804G cells as previously described in the work of [71]. Briefly, 2.5 × 10^5^ cells were spread onto a laminin 5-enriched matrix for 20 min. Images were obtained using a phase-contrast microscope (AXIO microscope with Axio Cam Erc5s, Zeiss-objective 10×, Heidelberg, Germany) at time 0 and after 20 min. Adherent cells on the laminin-enriched matrix were counted using NIH Image J 1.8 software (National Institutes of Health, Bethesda, MD, USA). Data were represented as percentage of adherent cells on laminin-enriched matrix.

### 4.12. Boyden’s Chamber Assay

Chemoinvasion assays were performed as in the work of [73] with minor modifications. Briefly, 2 × 10^4^ cells, after 48 h pre-treatment with E_2_ and/CoCl_2_ 100 µM, were placed in the upper compartment of the Boyden’s chamber, in the presence or absence of E_2_ and/or CoCl_2_ and/or EZH2/JMJD3 inhibitors, and after 16 h of incubation at 37 °C, invaded cells were fixed, stained with DiffQuick (Medion Diagnostics, Dudingen, Switzerland), and counted. Each assay was performed in quadruplicate and repeated at least three times. Images were obtained by Microscope OLYMPUS BX53 under 20× magnification.

### 4.13. Organotypic Slice Cultures (OSCs) from Prostate Cancer Specimens

This study was authorized by the ethical committee of IRCCS-Fondazione Policlinico Gemelli-Università Cattolica in Rome, Italy (IRCCS-Fondazione Policlinico Gemelli-Università Cattolica Protocol number: 232918/18; ID:2133, date 21 June 2018) and informed consent was obtained from each patient. All procedures were conducted according to the principles expressed in the Declaration of Helsinki, the institutional regulation, and Italian laws and guidelines. PCa patients (*n* = 10) were enrolled at the Department of Urology, Università Cattolica to perform prostatectomy with the following inclusion criteria: (i) clinically localized PCa at diagnosis and (ii) absence of hormone treatment/radiotherapy before surgery. OSCs were generated as described in the work of [40]. Medium was replaced daily and after three days, slices were treated with E_2_ (10^−7^ M) or CoCl_2_ (300 µM) alone or in combination, for the times indicated in figure legends, and then RNA and protein was extracted using Trizol according protocol described in the work of [74].

### 4.14. Statistical Analysis

Data are expressed as mean ± SEM or as fold induction as indicated in figure legends. Significance was calculated using a two-tailed *t*-test and/or one-way analysis of variance (ANOVA). Statistical analysis was performed using Sigma Plot 13.0 statistical software. *p* values of <0.05 were considered as significant.

## 5. Conclusions

Our data contribute elucidating the molecular basis underlying the acquisition of an aggressive phenotype in PCa under combined estrogen and hypoxia stimuli, focusing on H19 modulation and its impact on PCa biology. In particular, we demonstrated an important change in cell adhesion molecules, which involves β3 and β4 integrin subunits triggered by H19 under combined treatment. We revealed that H19 acts as a repressor of transcription of E-cadherin, as well as β3 and β4 integrins, by recruiting EZH2 polycomb subunit and increasing H3K27me3 level at the promoter region. Under combined treatment, we observed, at least in aggressive prostate cancer, a specific inhibition of H19 expression that leads to the release of cell adhesion molecules transcription. This phenomenon is crucial for the acquisition of aggressive and metastatic phenotype. Lastly, we demonstrated that epigenetic modulation of H3K27me3 level may restore the basal level of β3 and β4 integrins, thus reducing invasion capability. Finally, the effect of epigenetic drugs on the novel H19/integrin pathway might represent an alternative/additional tool to modulate cell adhesion and invasion. This may impact the metastatic potential and may be used to design innovative targeted therapies.

## Figures and Tables

**Table 1:** Clinical and pathologic feature of patients and tumors. PCa, prostate cancer; PSA, prostate-specific antigen.

PCa Patients	Age	PSA (ng/mL)	Pathologic Gleason Score	Pathologic Stage
**A**	61	8.5	7 (4 + 3)	pT2c pNx pMx
**B**	63	5.36	7 (3 + 4)	pT2c pNx pMx
**C**	70	18.04	7 (3 + 4)	pT2c pN0 pMx
**D**	68	5.75	7 (4 + 3)	pT3b pN0 pMx
**E**	55	6	7 (4 + 3)	pT3b pN0 pMx
**H**	78	15	7 (4 + 3)	pT3a pNx pMx
**I**	67	13.5	7 (4 + 3)	pT2c pN0 pMx
**L**	66	8.5	7 (4 + 3)	pT3b pN0 pMx

## Supplementary Materials

Supplementary materials can be found at https://www.mdpi.com/1422-0067/20/16/4012/s1.

## Abbreviations

PCa	prostate cancer
PSA	prostate-specific antigen
RP	radical prostatectomy
RT	radiation therapy
ECM	extracellular matrix
CDH1	E-cadherin
EMT	epithelial to mesenchymal transition
lncRNAs	long non-coding RNAs
ER	estrogen receptor
eNOS	endothelial nitric oxide synthase
HIFs	hypoxia inducible factors
HOTS	H19 Opposite Tumor Suppressor
E_2_	17β-estradiol
HYP	hypoxia
CoCl_2_	cobalt chloride
qPCR	quantitative polymerase chain reaction
KDR	vascular endothelial growth factor receptor 2
EPO	erythropoietin
ChIP	chromatin immunoprecipitation
TSS	transcriptional start site
HDAC	histone deacetylases
VHL	von Hippel-Lindau tumor suppressor
Ca^2+^	calcium
ITGB3	β3 integrin subunit
ITGB4	β4 integrin subunit
ITGA2	α2 integrin subunit
RUNX2	Runt-related transcription factor 2
H3K27me3	trimethylated lysine 27 of histone H3
OSCs	organotypic slice cultures
CCND1	Cyclin D1
RNA-ChIP	RNA chromatin immunoprecipitation
DMSO	dimethyl sulfoxide
EDTA	ethylenediaminetetraacetic acid
PBS	phosphate-buffered saline
