# Supplementary material for: H19-Dependent Transcriptional Regulation of β3 and β4 Integrins Upon Estrogen and Hypoxia Favors Metastatic Potential in Prostate Cancer
Source: Int J Mol Sci. 2019 Aug 17;20(16):4012. doi: 10.3390/ijms20164012 (PMC6720303; doi:10.3390/ijms20164012)
Supplement: Supplementary file 1 [file ijms-20-04012-s001.pdf]

a

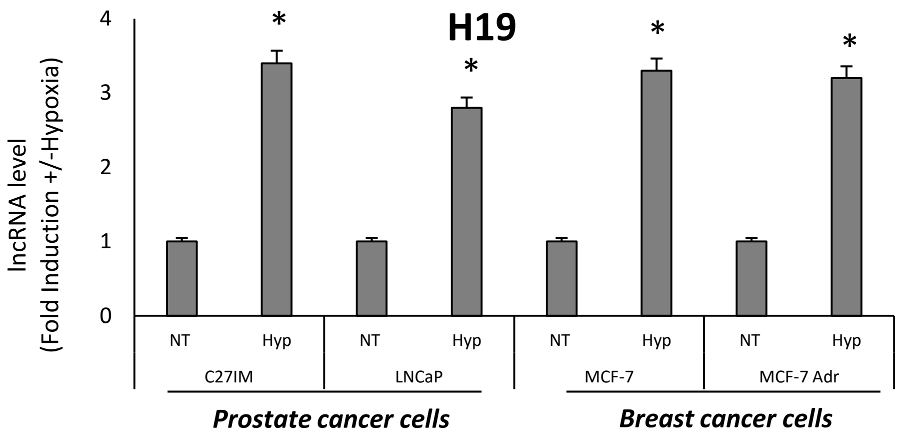

b

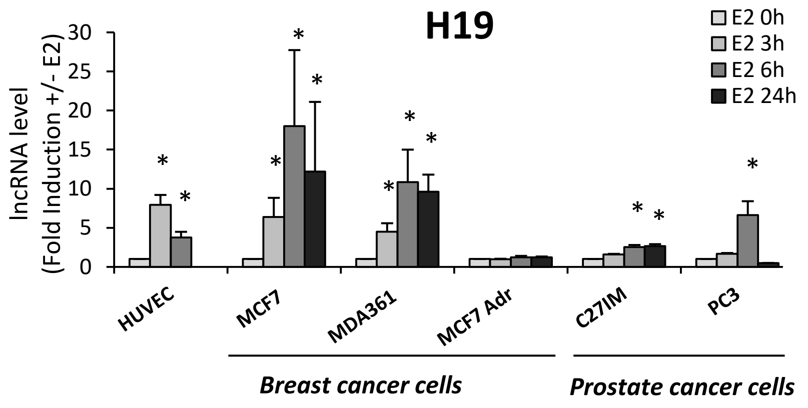

Figure S1. Estrogen- or hypoxia-dependent H19 expression in prostate and breast cancer cells. a) H19 level was assessed by quantitative RT-PCR in Prostate cancer (C27IM and LNCaP) and in Breast cancer (MCF-7, MCF-7 Adr) cells in normoxia (NT) or hypoxia (Hyp, 1% O_2, 18 hours). Data, plotted as fold induction vs NT, represent mean ± SEM of 3 experiments. *p<0,05 vs NT. b) H19 level was assessed by quantitative RT-PCR in normal human endothelial cell line HUVEC, Breast cancer (MCF-7, MDA361, MCF7 Adr) and in Prostate cancer (C27IM and PC3) cells in basal condition (E2 0h) and at 3h, 6h and 24h upon treatment with E2 (10^{-7}M). Data, plotted as fold induction, represent mean ± SEM of 3 experiments. *p<0,05 vs NT.

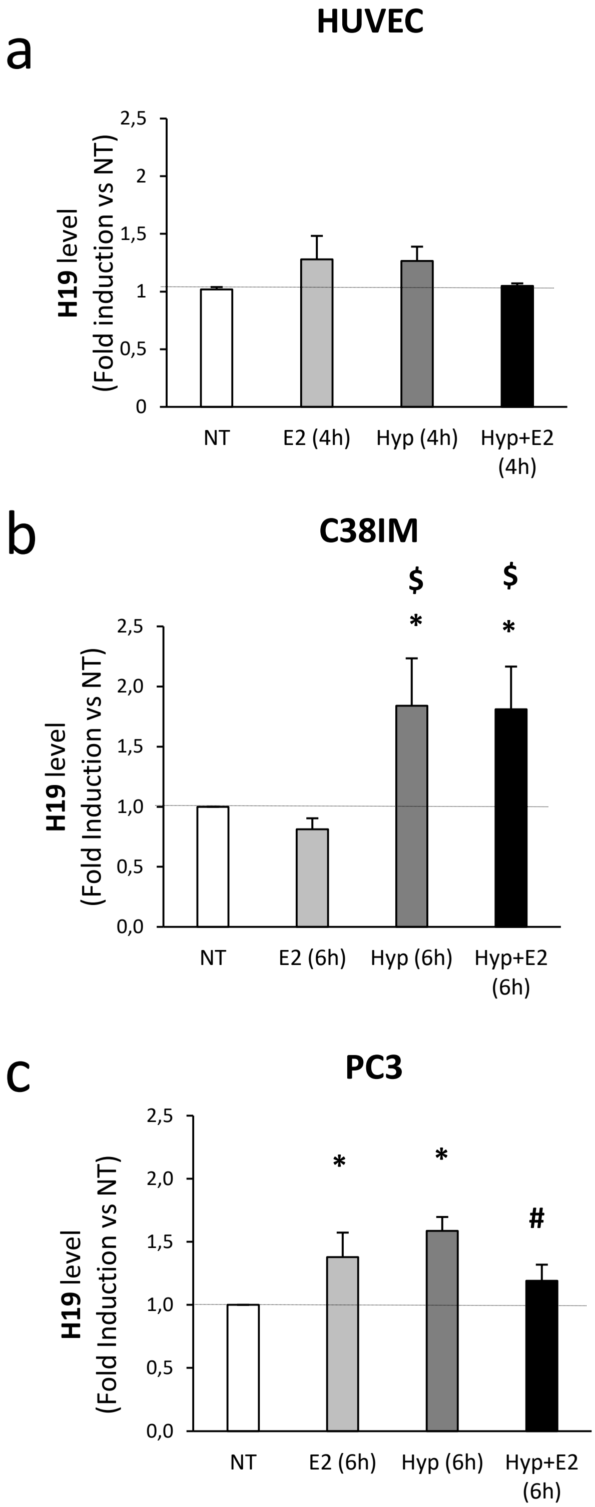

Figure S2. H19 regulation by hypoxia and estrogen treatment in normal and transformed cell lines. H19 level was assessed in normal cells (HUVEC, a), in cells derived from clinically-localized prostate cancer (C38IM, b) and from prostate cancer metastasis-derived cell line (PC3, c) by quantitative RT-PCR after 4 or 6 hours treatment with E2 (10^{-7}M) and 1% O_2 hypoxia (Hyp), alone or in combination. Data, plotted as fold induction, represent mean ± SEM of 3 experiments. *p<0,05 vs NT; $ p<0,05 vs E2; # p<0,05 vs Hyp.

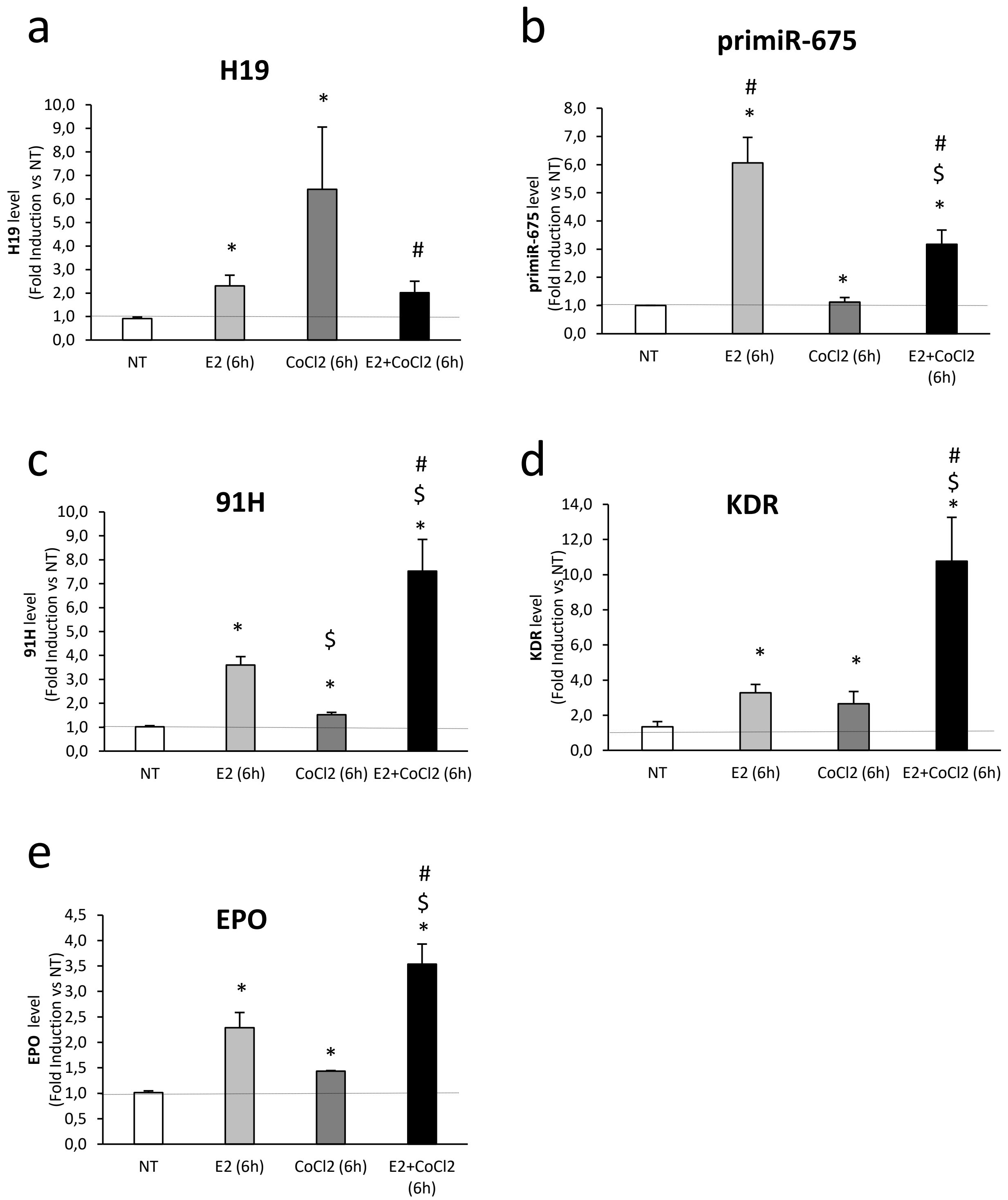

Figure S3. Opposite transcripts of H19 gene locus are differentially regulated after chemical hypoxia and estrogen treatment while combined treatment have a synergistic effect on the classical hypoxia target gene. H19 (a), primiR-675 (b), 91H (c), KDR (d) and EPO (e) levels were assessed by quantitative RT-PCR in C27IM after 6 hours treatment with E2 (10^{-7}M) and CoCl2 (100µM) alone or in combination. Data, plotted as fold induction, represent mean ± SEM of 3 experiments. *p<0,05 vs NT; $ p<0,05 vs E2; # p<0,05 vs CoCl2.

a

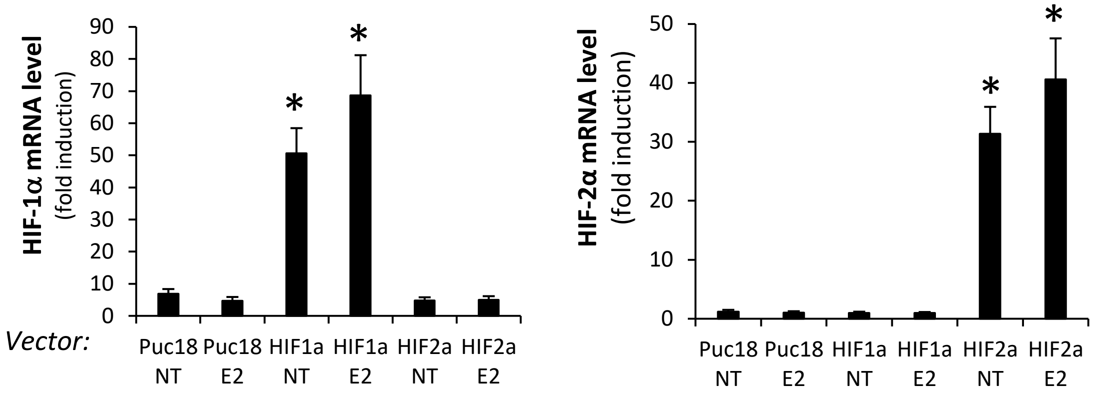

b

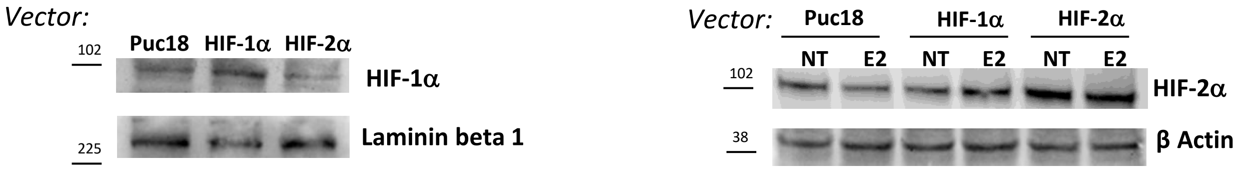

Figure S4. Evaluation of HIF-1α and HIF-2α overexpression in C27IM cells. a-b) C27IM cells were transfected for 72h with HIF-1α or HIF-2α expression vectors. The empty vector Puc18 (Puc18) was used as negative control. a) HIF-1α and HIF-2α mRNA levels were quantified by qPCR in presence or absence of E2 (10^{-7}M; 6 hours) using primers specific to HIF-1α (left) or HIF-2α (right). Data, plotted as fold induction vs NT, represent mean ± SEM of 3 experiments. *p<0,05 vs Puc18; b) Western blot of HIF-1α (left) or HIF-2α (right). β Actin or Laminin beta 1 served as loading control. Molecular weight marker is indicated.

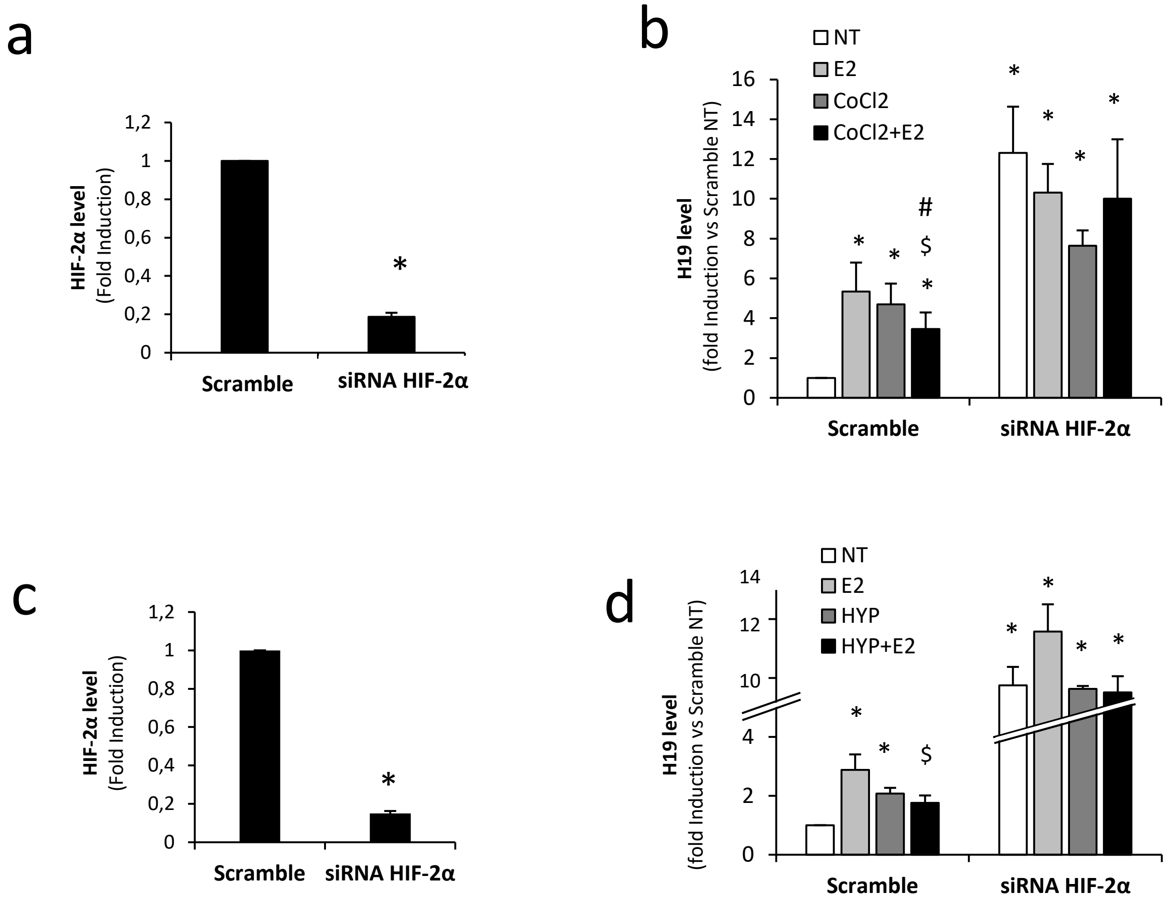

Figure S5. Effect of HIF-2α silencing on H19 expression. a and c) HIF-2α RNA levels quantified by qPCR in 786-O or C27IM respectively, transfected with siRNA specific to HIF-2α (siRNA HIF-2α) or scramble. Data, plotted as fold induction siRNA HIF-2α vs scramble, represent mean ± SEM of 3 experiments. *p<0,05 vs Scramble. b and d) H19 RNA levels quantified by qPCR in 786-O or C27IM transfected with siRNA specific to HIF-2α (siRNA HIF-2α) or scramble and treated with estrogen (E2, 10^{-7}M) or hypoxia (CoCl2 or HYP), alone or in combination (CoCl2+E2) for 6h. Data, plotted as fold induction siRNA HIF-2α vs scramble NT, represent mean ± SEM of 3 experiments. *p<0,05 vs NT scramble; $ p<0,05 vs E2 scramble; # p<0,05 vs CoCl2 scramble.

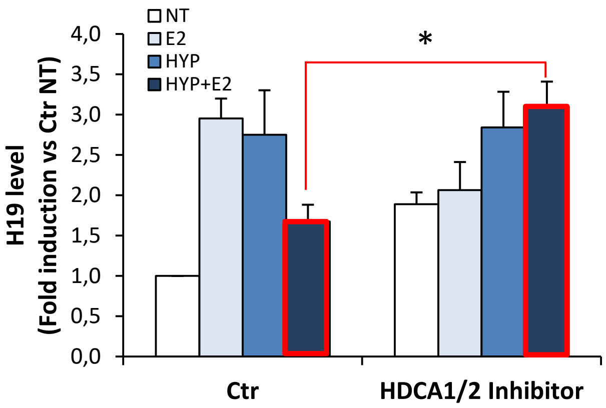

Figure S6. Effects of HDAC1/2 inhibitor on H19 expression under combined treatment. H19 levels assessed by qPCR in C27IM after 6 hours treatment with E2 or HYP, alone or in combination, in the presence or absence of inhibitor specific to HDAC1/2 (Mocetinostat2HBr, 1µM) added 30 min before E2 and/or HYP. Data, plotted as fold induction vs NT/Ctr, represent mean ± SEM of 3 experiments. *p<0,05.

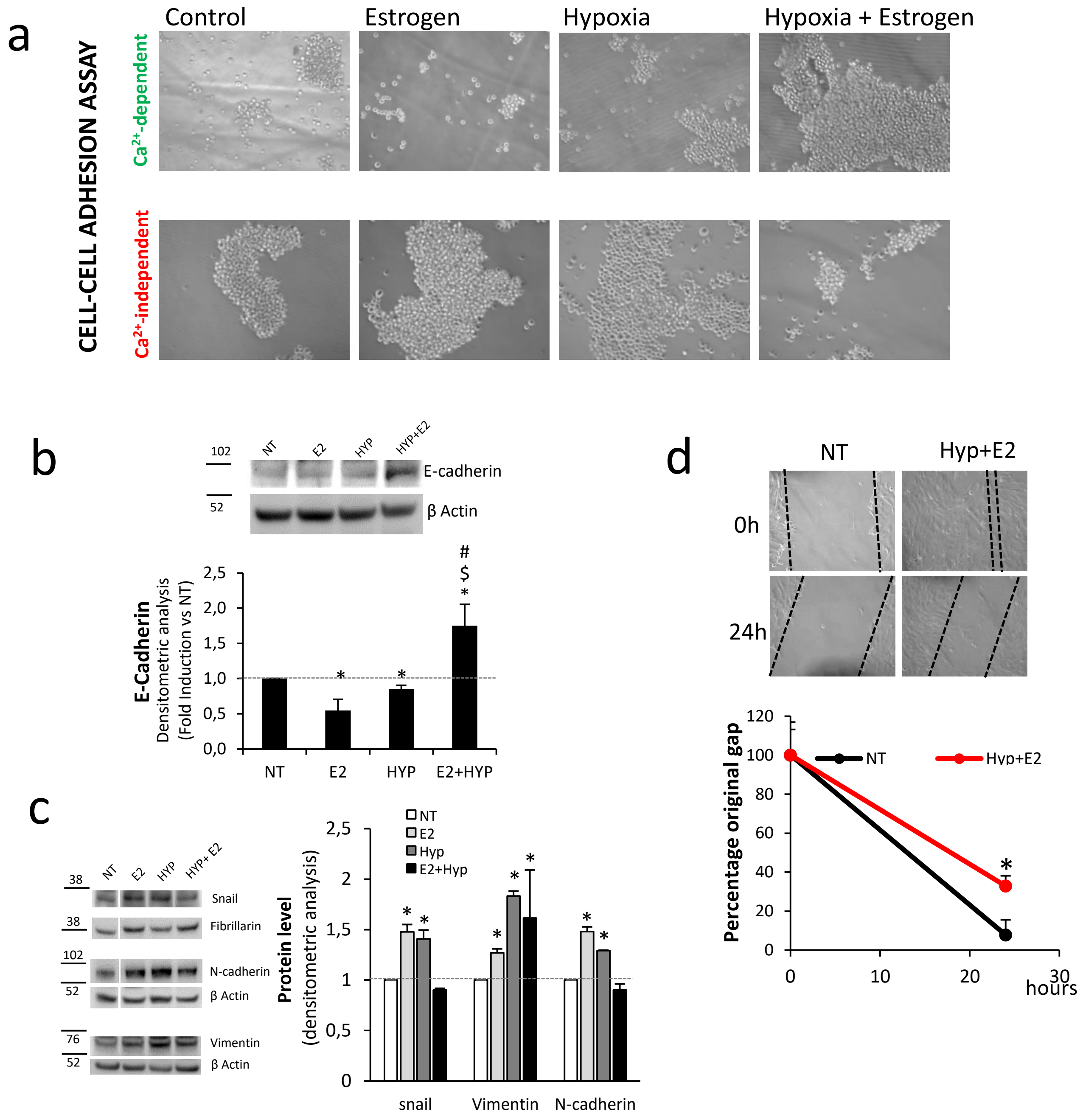

Figure S7. Cell to cell adhesion assay, expression of E-cadherin and EMT-related genes and scratch-test upon estrogen and hypoxia. a) Representative phase contrast images (bright field) of cell-cell adhesion assay performed in presence (Ca^{2+}-dependent) or in absence (Ca^{2+}-independent) of 1mM Ca^{2+} as in Figure 3a and b. b-d) Expression of EMT-related genes after 18 hours treatment with E_2 (10^{-7}M) or 1%O_2 hypoxia (HYP), alone or in combination. b) Protein level analysis of E-cadherin in C27IM by western blot. β Actin was used as control. Molecular weight marker is indicated. Upper panel: representative experiment. Lower panel: densitometric analysis, reported as fold induction vs NT, represent mean ± SEM of 3 experiments. *p<0,05 vs NT, $ p<0,05 vs E2, # p<0,05 vs HYP. c) Protein level analysis of EMT-related genes, the E-cadherin transcriptional repressor SNAIL (on nuclear extracts), Vimentin and N-cadherin (on cytoplasmic extracts) in C27IM by western blot. Fibrillarin or β Actin were used as control. Molecular weight marker is indicated. Left panels: representative experiments. Right panels: densitometric analysis, reported as fold induction vs NT, represent mean ± SEM of 3 experiments. *p<0,05 vs NT. White bars indicate non contiguous lines of same gel. d) Representative phase contrast images in bright field of a scratch test (upper panel) and its quantification (lower panel) before and after 24 hours under normoxia (NT) or combined treatment estrogen (E_2, 10^{-7} M) plus 1% O_2 hypoxia (Hyp+E2). Results are expressed as percentage of original gap. Data represent mean ± SEM of 3 independent experiments. * p<0,05 vs NT.

a

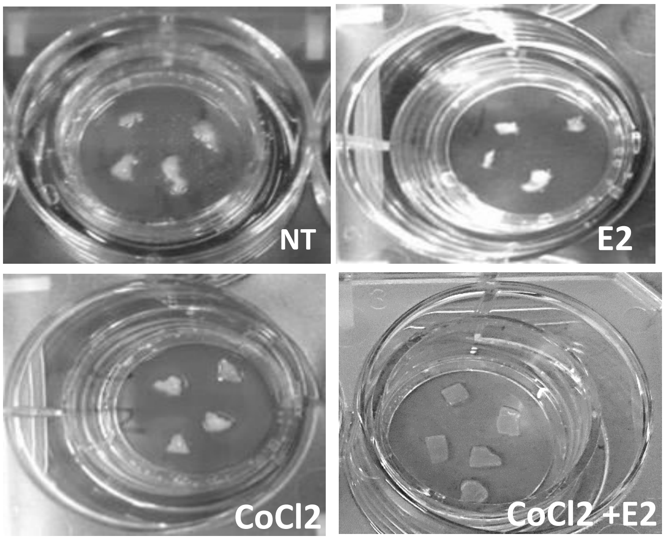

b

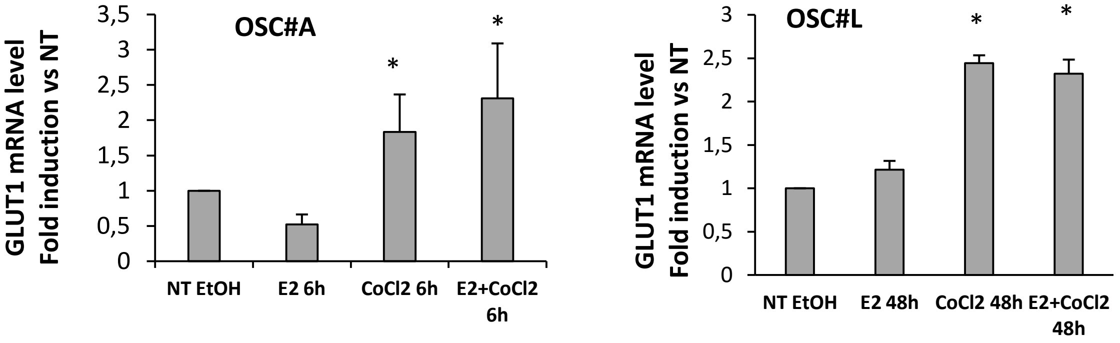

c

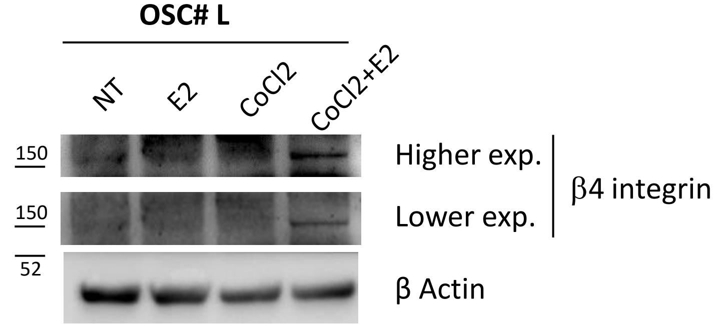

Figure S8. ***Ex vivo*** **Organotypic Slice Cultures (OSCs) of prostate tumors, GLUT1 and β4 integrin expression under combined treatment.** a) Representative images of set of Organotypic Slices (OSCs) after 3 days. OSCs were treated with 17β-estradiol (10^{-7}M, E2) or CoCl2 (300µM) alone or in combination before harvesting. b) GLUT1 mRNA levels quantified by qRT-PCR in PCa-derived OSCs after 6 hours (left) or 48 hours (right) treatment with E2 and/or CoCl2, alone or in combination. Data, plotted as fold induction vs NT, shows a representative OSC. * p<0,05 vs NT. c) β4 integrin protein level assessed in OSC#10 after 48 hours treatment with E2 and/or CoCl2 alone or in combination performed by western blot. Two different exposure (Higher and Lower exp.) are showed. β Actin was used as control. Molecular weight marker is indicated.

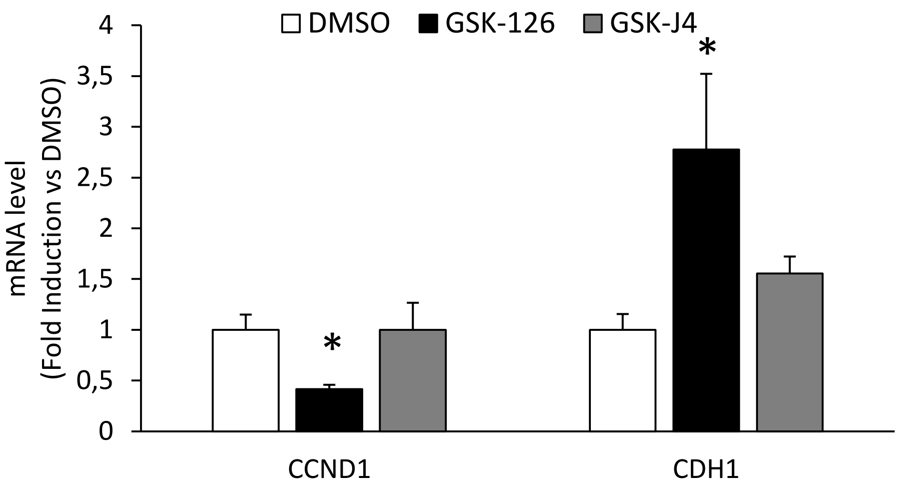

Figure S9. Modulation of EZH2-taget genes E-cadherin and cyclin D1 upon EZH2 or JMJD3 inhibitors. Cyclin D1 (CCND1) and E-cadherin (CDH1) levels were assessed by qPCR in C27IM after 18 hours treatment with estrogen (E2; 10^{-7}M), 1% O_2 hypoxia (HYP), alone or in combination, in the presence or absence of inhibitors to EZH2 (GSK-126, 1µM) or JMJD3 (GSK-J4, 1µM) added 30 min before E2 and/or HYP. Data, plotted as fold induction vs DMSO, represent mean ± SEM of 3 experiments. * p<0,05 vs DMSO.

Bacci et al. IJMS #582123 _Figure S4b uncropped

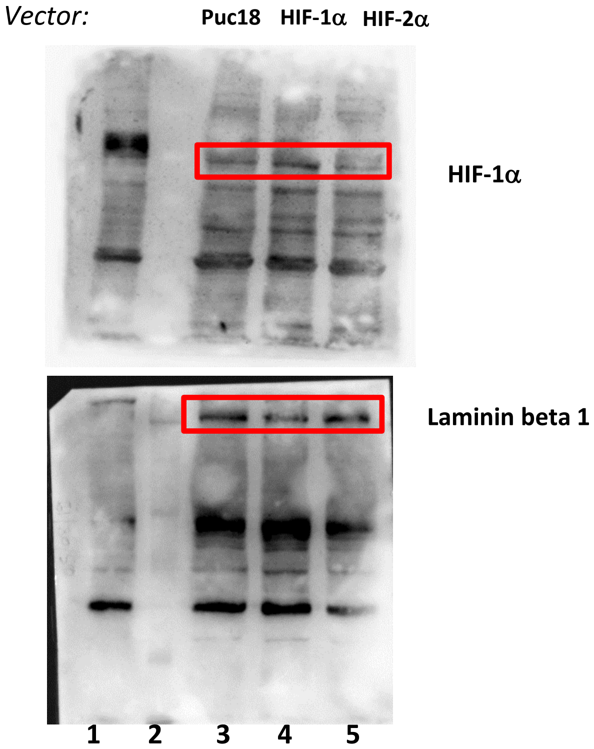

Gel #1 (left panel)

Uncut gels related to WB depicted in Supplementary Figure 4b, left panel. Whole membrane blots of western blot analysis (7.5 % acrylamide gel) related to HIF-1α (upper) and Laminin beta1 (lower) in PCa cells (C27IM, lanes 3-5, 40μg) nuclear extracts before/after overexpression of HIF1α or HIF2α, as indicated. Lane 1 is loaded with C27IM treated with CoCl2 as positive control for HIF-1α (10μg). Lane 2 is loaded with MW marker. Images of selected portions shown in the original Figure S4b are highlighted in red.

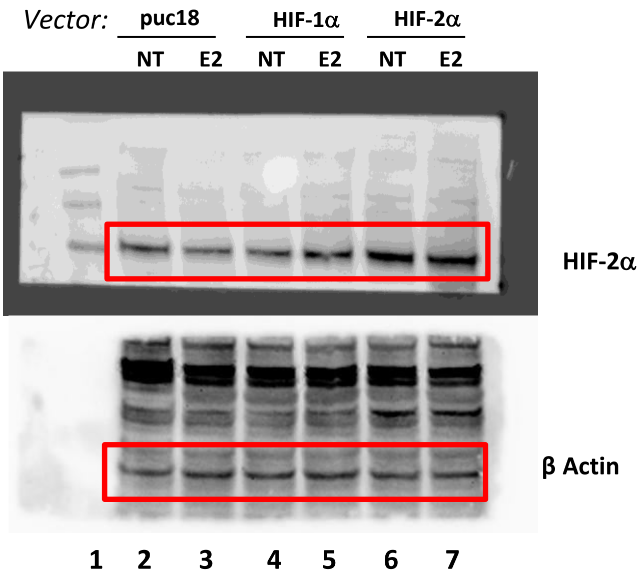

Gel #2 (right panel)

Uncut gels related to WB depicted in Supplementary Figure 4b, right panel. Whole membrane blots of western blot analysis (4-12% acrylamide gel) related to HIF-2α (upper) and β-actin (lower) in PCa cells (C27IM, lanes 2-7) total lysates before/after overexpression of HIF1α or HIF2α, as indicated. Lane 1 is loaded with MW marker. Images of selected portions shown in the original Figure S4b are highlighted in red.

Bacci et al. IJMS #582123 _Figure S7b uncropped

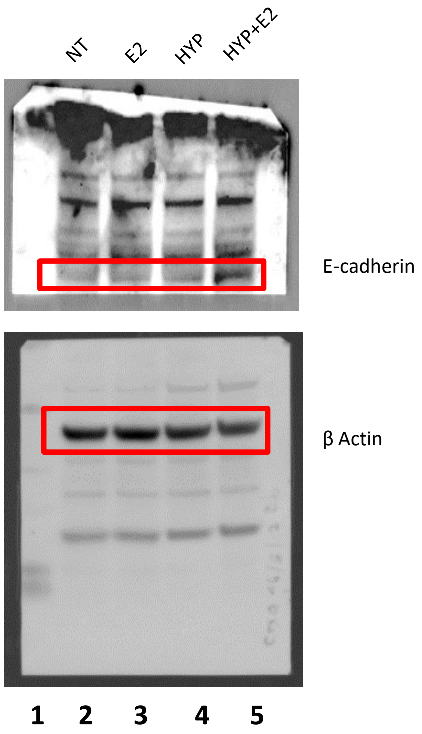

Uncut gels related to WB depicted in Supplementary Figure 7b. Whole membrane blots of western blot analysis (4-12 % acrylamide gel) related to E-cadherin (upper) and β Actin (lower) in PCa cells (C27IM, lanes 2-5) cytosolic extracts in untreated or upon E2, Hypoxia, or E2+Hypoxia treatment as indicated in the Figure S7 Legend. Lane 1 is loaded with MW marker. Images of selected portions shown in the original Figure S7b are highlighted in red.

Bacci et al. IJMS #582123_Figure S7c uncropped

Gel #1 (upper panel)

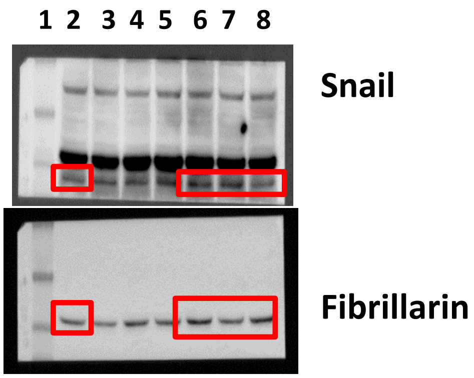

Uncut gels related to WB depicted in Supplementary Figure 7c. Whole membrane blots of western blot analysis (4-12 % acrylamide gel) related to Snail (upper) and Fibrillarin (lower) in PCa cells (C27IM, lanes 2-8) nuclear extracts in untreated or upon E2, Hypoxia, or E2+Hypoxia treatment as indicated in the Figure S7 Legend. Lane 1 is loaded with MW marker. Images of selected portions shown in the original Figure S7c are highlighted in red.

Gel #2 (middle panel)

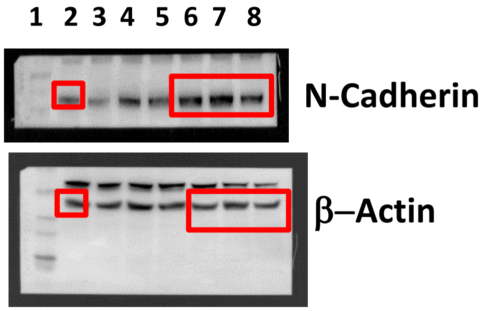

Uncut gels related to WB depicted in Supplementary Figure 7c. Whole membrane blots of western blot analysis (4-12 % acrylamide gel) related to N-Cadherin (upper) and β-Actin (lower) in PCa cells (C27IM, lanes 2-8) cytosolic extracts in untreated or upon E2, Hypoxia, or E2+Hypoxia treatment as indicated in the Figure S7Legend. Lane 1 is loaded with MW marker. Images of selected portions shown in the original Figure S7c are highlighted in red.

Bacci et al. IJMS #582123 _Figure S7c uncropped *(continued)*

Gel # 3 (lower panel)

1 2 3 4 5 6 7 8

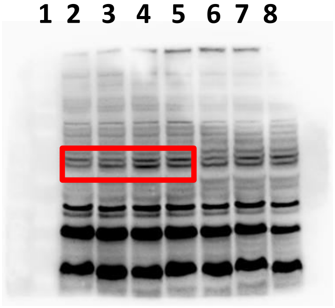

Vimentin

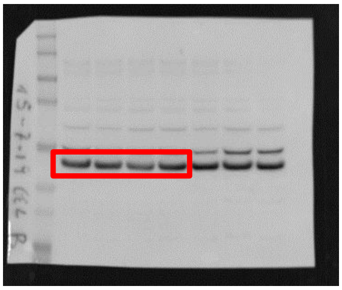

β–Actin

Uncut gels related to WB depicted in Supplementary Figure 7c. Whole membrane blots of western blot analysis (4-12 % acrylamide gel) related to Vimentin (upper) and β-Actin (lower) in PCa cells (C27IM, lanes 2-8) cytosolic extracts in untreated or upon E2, Hypoxia, or E2+Hypoxia treatment as indicated in the Figure S7 Legend. Lane 1 is loaded with MW marker. Images of selected portions shown in the original Figure S7c are highlighted in red.

Bacci et al. IJMS #582123_Figure S8c uncropped

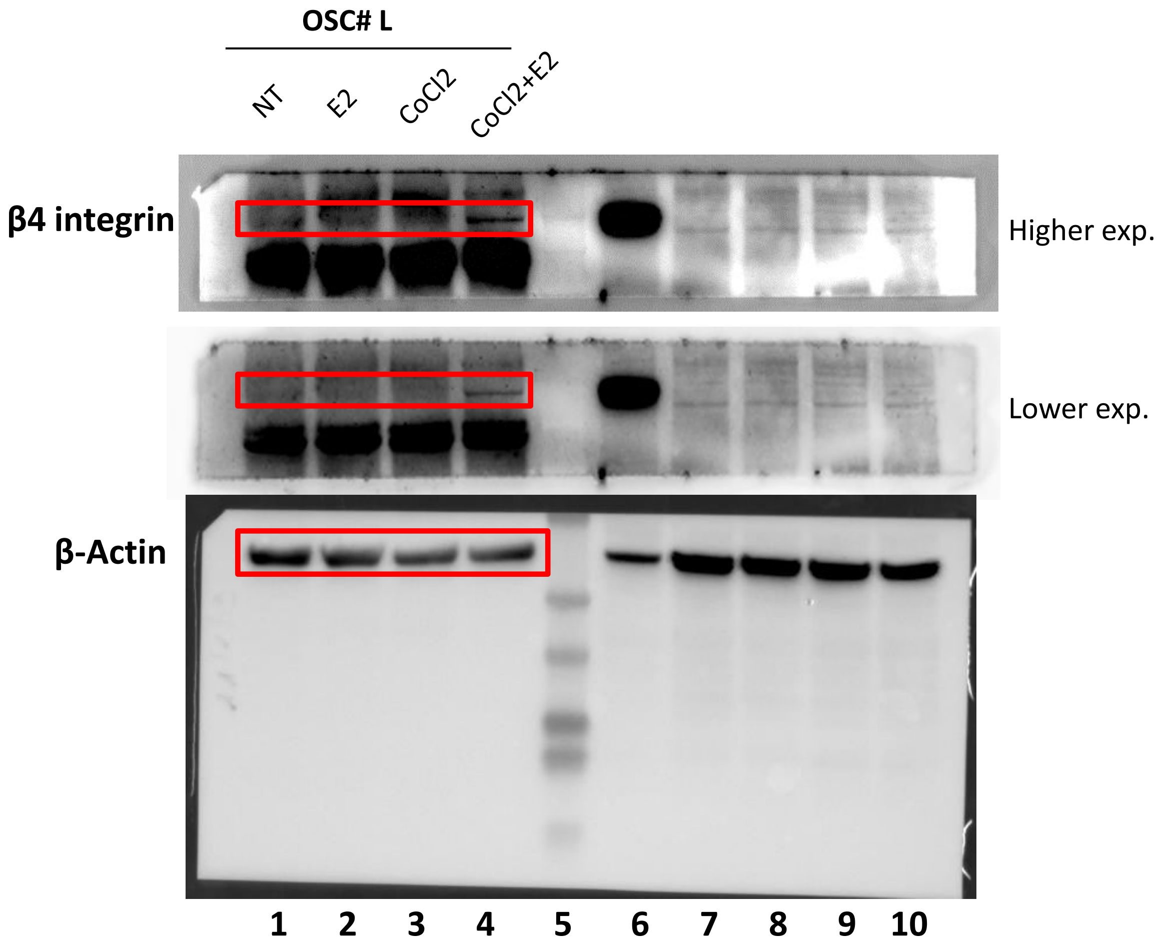

Uncut gels related to WB depicted in Supplementary Figure 8c. Whole membrane blots of western blot analysis (4-12 % acrylamide gel) related to N-Cadherin (upper) and β-Actin (lower) in Organotypic Slice Culture #L (lanes 1-4) whole tissue extracts before or after E2, Hypoxia, or E2+Hypoxia treatment as indicated in the S8 Figure Legend. Lane 5 is loaded with MW marker and lane 6 with MCF7 as positive control. Images of selected portions shown in the original Figure S8c are highlighted in red.

Bacci et al. IJMS #582123_Figure 3e uncropped

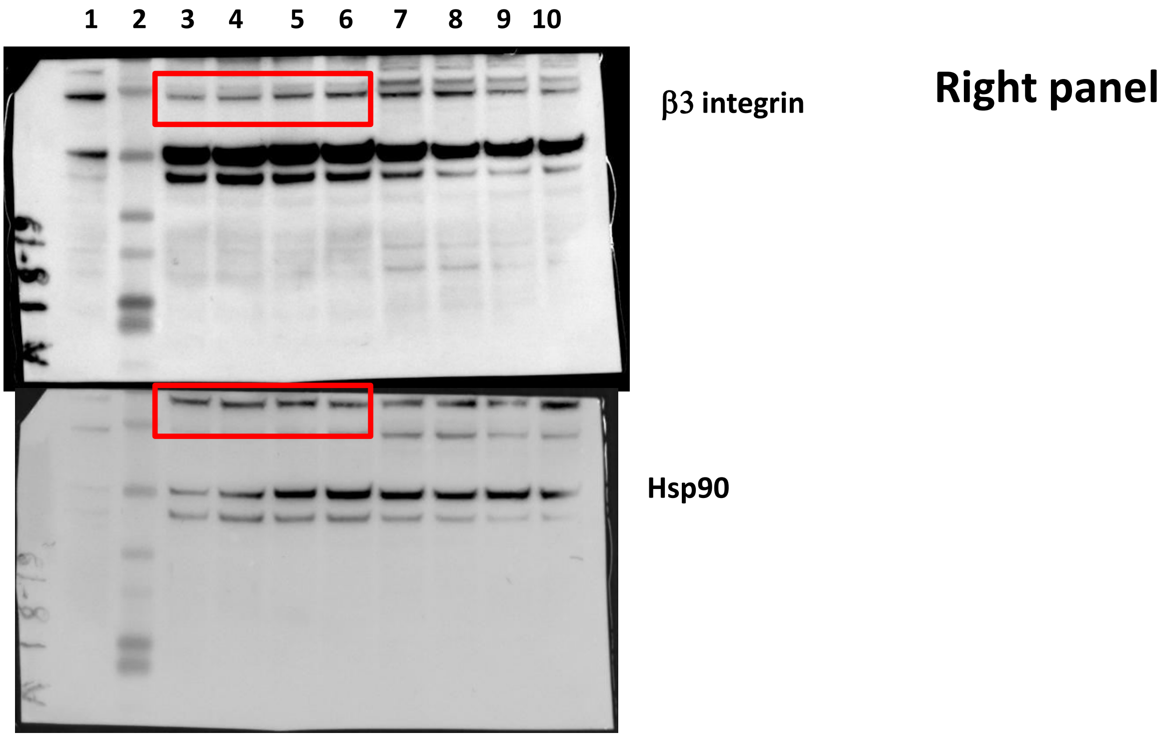

Uncut gels related to WB depicted in Figure 3e. Whole membrane blots of western blot analysis (4-12% acrylamide gel) related to β3 integrin (upper) and Hsp90 (lower) in MCF7 (lane 1) or PCa cells (C27IM, lanes 3-10) cytosolic lysates in untreated or upon E2, Hypoxia, or E2+Hypoxia treatment as indicated in the Figure 3. Lane 2 is loaded with MW marker. Images of selected portions shown in the original Figure 3e are highlighted in red.

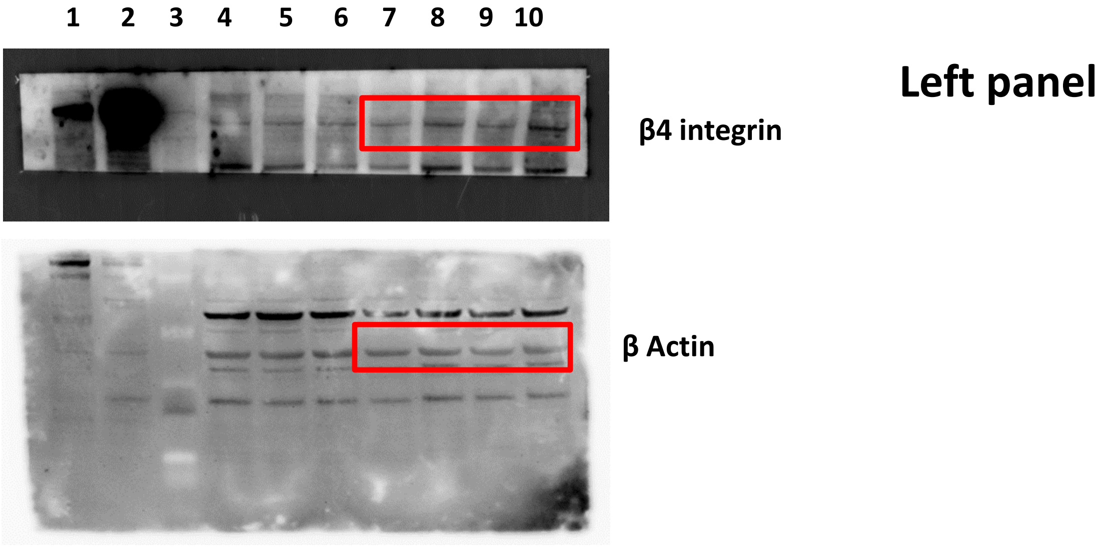

Uncut gels related to WB depicted in Figure 3e. Whole membrane blots of western blot analysis (4-12% acrylamide gel) related to β4 integrin (upper) and β Actin (lower) in MCF7 or in β4 integrin positive control (lane 1-2) or PCa cells C27IM (lanes 4-10) cytosolic lysates in untreated or upon E2, Hypoxia, or E2+Hypoxia treatment as indicated in the Figure 3. Lanes 3 is loaded with MW marker. Images of selected portions shown in the original Figure 3e are highlighted in red.

Bacci et al. IJMS #582123_Figure 4d uncropped

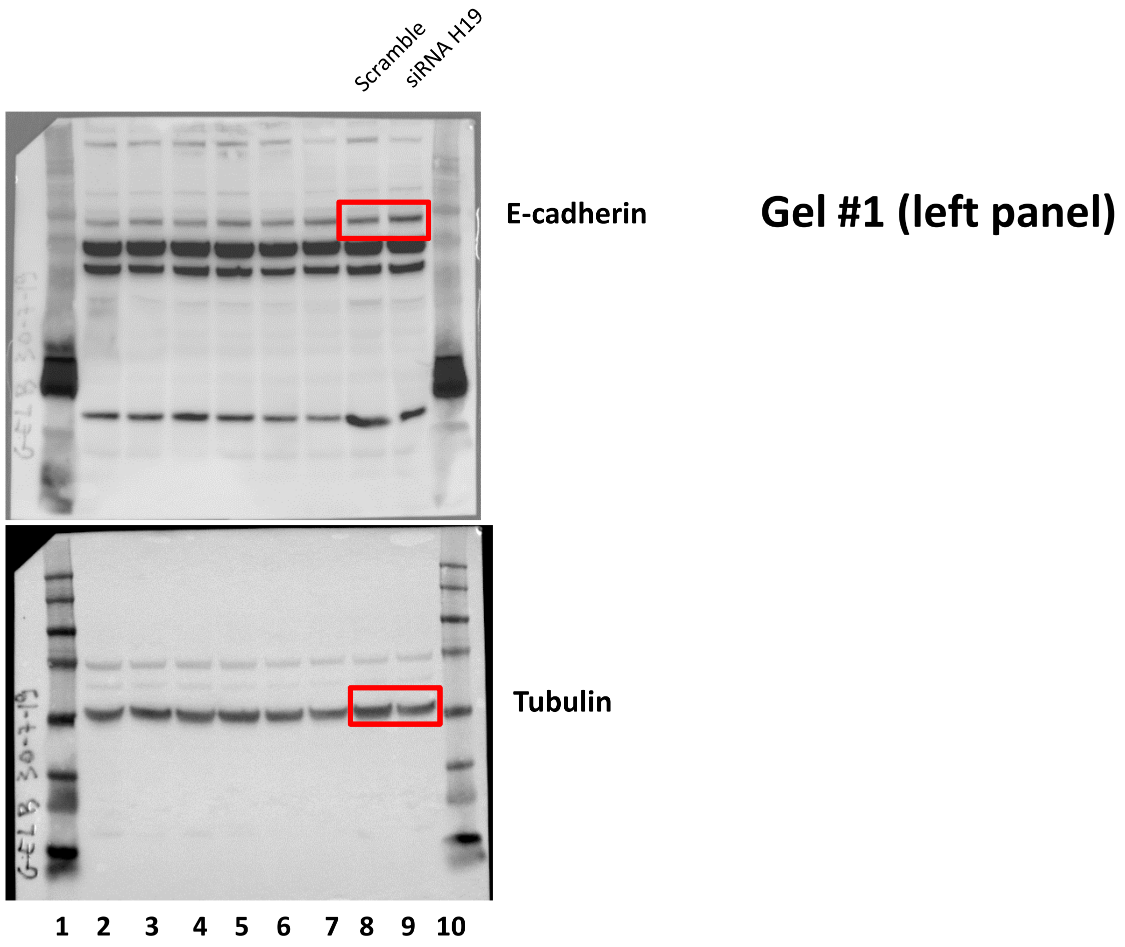

Uncut gels related to WB depicted in Figure 4d (left panel). Whole membrane blots of western blot analysis (4-12% acrylamide gel) related to E-cadherin (upper) and Tubulin (lower) in PCa cells C27IM (lanes 2-9) cytosolic lysates. Lanes 1 and 10 is loaded with MW marker. Images of selected portions, lanes 8 and 9, shown in the original Figure 4d are highlighted in red.

Bacci et al. IJMS #582123_Figure 4d uncropped

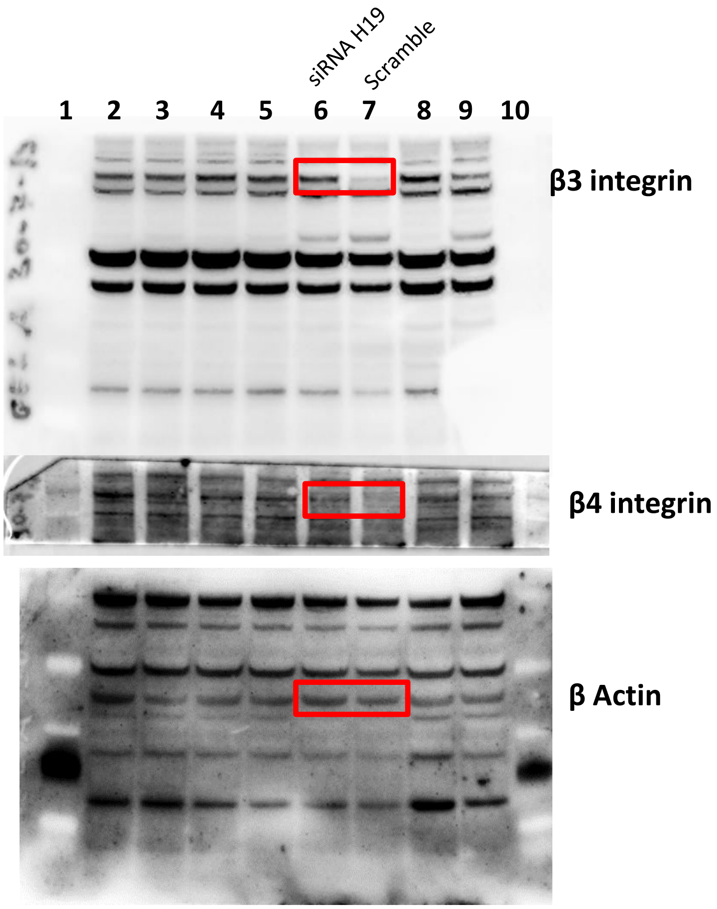

Gel #2 (right panel)

Uncut gels related to WB depicted in Figure 4d (right panel). Whole membrane blots of western blot analysis (4-12% acrylamide gel) related to β3 integrin (upper), β4 integrin (middle) and β Actin (lower) in PCa cells C27IM (lanes 2-9) cytosolic lysates. Lanes 1 and 10 is loaded with MW marker. Images of selected portions, lanes 6 and 7, shown in the original Figure 4d are highlighted in red.
